# The Impact of a Very Short Abstinence Period on Conventional Sperm Parameters and Sperm DNA Fragmentation: A Systematic Review and Meta-Analysis

**DOI:** 10.3390/jcm11247303

**Published:** 2022-12-08

**Authors:** Federica Barbagallo, Rossella Cannarella, Andrea Crafa, Claudio Manna, Sandro La Vignera, Rosita A. Condorelli, Aldo E. Calogero

**Affiliations:** 1 Department of Clinical and Experimental Medicine, University of Catania, 95124 Catania, Italy; 2 Biofertility IVF and Infertility Center, 00198 Rome, Italy; 3 Department of Biomedicine and Prevention, University of Rome “Tor Vergata”, 00133 Rome, Italy

**Keywords:** ejaculatory abstinence, sexual abstinence period, consecutive ejaculation, sperm DNA fragmentation, couples infertility

## Abstract

Purpose: In recent years, a growing number of studies have supported the beneficial effects of a very short abstinence period on sperm parameters, especially in patients with oligoasthenozoospermia. However, the results are controversial and no consensus exists regarding whether to request a second semen collection in clinical practice. Therefore, this systematic review and meta-analysis aimed to evaluate the influence of a very short abstinence period (within 4 h) on conventional sperm parameters and sperm DNA fragmentation (SDF) rate. Materials and Methods: The literature search was performed using Scopus and PubMed databases. The meta-analysis was conducted according to the Preferred Reporting Items for Systematic Review and Meta-Analysis Protocol (PRISMA-P) guidelines. All eligible studies were selected according to the Population, Intervention, Comparison/Comparator, Outcomes, and Study design (PICOS) model. The quality of evidence of the included studies was analyzed through the Cambridge Quality Checklists. The standardized mean difference (SMD) was used to analyze the outcomes. Cochran-Q and I^2^ statistics were used to evaluate statistical heterogeneity. Results: We assessed for eligibility 1334 abstracts, and 19 studies were finally included. All 19 articles evaluated the effects of a very short abstinence period on sperm parameters and, among these, 5 articles also evaluated the effects on SDF rate. The quantitative analysis showed a significant reduction in semen volume after a very short abstinence period in both normozoospermic men and patients with oligozoospermia, asthenozoospermia, and/or teratozoospermia (OAT) patients. We found a statistically significant increase in sperm concentration and total and progressive motility in the second ejaculation of patients with OAT. In contrast, the SDF rate decreased significantly in the second ejaculate of OAT patients. Conclusions: This is the first systematic review and meta-analysis investigating the impact of a very short abstinence period on sperm parameters and SDF rate. The results suggest that collecting a second consecutive ejaculation after a very short time from the first could represent a simple and useful strategy for obtaining better-quality spermatozoa, especially in patients with abnormal sperm parameters.

## 1. Introduction

The World Health Organization (WHO) defines infertility as the inability to conceive after at least 12 months of regular, unprotected sexual intercourse [1]. Infertility remains a global public health issue, affecting approximately 8–12% of couples of reproductive age [2]. The male factor is responsible for couples’ infertility in about half of the cases [3]. Several causes contribute to the increasing prevalence of male infertility, which may be related to congenital, acquired, and idiopathic factors that impair spermatogenesis [3]. The causes of male infertility can be classified as factors acting at the pre-testicular, testicular, or post-testicular level. Nevertheless, despite several steps forward, male infertility remains a poorly understood area. In fact, to date, 50% of infertile patients have not received an etiological diagnosis and are defined as having idiopathic infertility [3]. Lifestyle and environmental factors, such as smoking [4], obesity [5], endocrine disruptors [6], exposure to heavy metals [7], or psychological stress [8], can play an important role in increasing the prevalence of male infertility.

Conventional sperm parameters (sperm concentration, motility, and morphology) are among the many predictors of male fertility and, to date, are still regarded as the cornerstone of fertility diagnosis despite the wide variability existing within and between men [9]. These variations may be attributed to several modifiable factors, including the latter and the length of sexual abstinence and the ejaculation frequency. Among these, sexual abstinence is often overlooked, although the length of sexual abstinence has been shown to influence sperm parameters. WHO laboratory manuals for the examination and processing human semen published since 1980 and the most recently released in 2021 [10] recommend that semen should be collected for semen analysis after a minimum of 2 days and a maximum of 7 days of sexual abstinence and this instruction has remained unchanged in all these years. However, the European Society of Human Reproduction and Embryology (ESHRE) recommends an abstinence period of only 3–4 days [11]. The basis for these recommendations is unclear and much evidence shows that a change in the current indications on the abstinence length is needed [12,13]. Many years ago, McLeod and Gold indicated that the period of abstinence should be based on the frequency of copulation [14]. They reported that a coital frequency of fewer than three times per week could result in delayed fertility due to a missing ovulatory window and/or impaired sperm parameters [14].

Several studies have investigated the influence of the length of sexual abstinence on sperm parameters, although the results are still controversial. Indeed, a longer abstinence period appears to improve semen fluid volume and sperm count whereas the effects on sperm motility, morphology, and DNA fragmentation (SDF) rate are still contradictory [15]. Furthermore, a growing number of studies have focused on the possibility to use a second ejaculation collected after a very short period of abstinence in infertile patients, especially in patients with oligoasthenozoospermia (OA). We previously reported that a second consecutive ejaculate (collected within 1 h from the first) resulted in better conventional sperm parameters (motility and morphology) and a lower percentage of spermatozoa with fragmented DNA in normozoospermic male partners of infertile couples and even more in patients with oligoasthenoteratozoospermia (OAT) [16]. Our findings were in line with the most recent literature [17,18,19,20,21,22]. However, no consensus exists on whether to request a second successive sample.

Therefore, this systematic review and meta-analysis aimed to evaluate the influence of a very short abstinence period on conventional sperm parameters and the SDF rate.

## 2. Materials and Methods

### 2.1. Sources

This study was performed by applying the Preferred Reporting Items for Systematic Review and Meta-Analysis Protocols (PRISMA-P) [23]. The PRISMA checklist is reported in Supplementary Table S1. The articles were selected through extensive searches in PubMed and Scopus databases from their establishment until June 2022. In detail, the following search string was used to search the Scopus database: TITLE-ABS-KEY (consecutive AND ejaculate) OR TITLE-ABS-KEY (consecutive AND ejaculation) OR TITLE-ABS-KEY (consecutive AND semen collection) OR TITLE-ABS-KEY (repeated AND ejaculate) OR TITLE-ABS-KEY (repeated AND ejaculation) OR TITLE-ABS-KEY (repeated AND semen collection) OR TITLE-ABS-KEY (second AND ejaculation) OR TITLE-ABS-KEY (second AND ejaculate). Additional manual searches were carried out using the reference lists of relevant studies. The search was limited to human studies and only English articles were selected. All abstracts and relevant full texts were evaluated. Two authors independently (F.B. and A.C.) reviewed the abstracts and selected only the articles that were pertinent to the objective of this study. Any disagreement was resolved by discussion with a third investigator (R.C.). The reference lists of the identified articles were also used to find pertinent studies.

### 2.2. Study Selection

All the eligible studies were selected following the PICOS (Population, Intervention, Comparison/Comparator, Outcomes, Study design) model (Table 1). We considered for inclusion all studies that evaluated the effects of a very short abstinence period (within 4 h) on sperm parameters (volume, concentration, total and progressive motility, and morphology) and SDF rate. Case reports, comments, letters to the editor, systematic or narrative reviews, and studies that did not allow extracting the outcomes of interest were excluded from the analysis.

### 2.3. Data Extraction

Data extraction was performed by one author (F.B.) and verified by a second one (A.C.). Disagreements were resolved by a third author (A.E.C.). The following data were collected: sperm conventional parameters (semen volume, sperm concentration, sperm progressive motility, sperm total motility, sperm morphology) and SDF of the first and second ejaculation, semen characteristics of patients enrolled (normozoospermic or OAT), abstinence period of first ejaculate, abstinence period of second ejaculate (within 4 h), and methods used for semen analysis and for the assessment of SDF.

### 2.4. Quality of Evidence

The quality assessment of the articles included in this systematic review and meta-analysis was performed using the “Cambridge Quality Checklists” [24]. This checklist comprises three domains designed to identify high-quality studies of correlates, risk factors, and causal risk factors. The checklist for correlates consists of five items that can be given a score of zero or one for a total of five. It evaluates the appropriateness of the sample size and the quality of the outcome measurements. The checklist for risk factors consists of three items; the selection of one of the three items excludes the other two, with a maximum score of 3 points. This checklist assigns high-quality scores only to those studies with appropriate time-ordered data. Finally, there is the checklist for causal risk factors that evaluates the type of study design, assigning the highest score to randomized clinical trials (RCTs) and the lowest score to cross-sectional studies without a control group. The maximum score is seven. To draw confident conclusions about correlates, the correlate score must be high. This means that the sample size must be large and the outcome assessment must be adequate and reproducible. To draw confident conclusions about risk factors, both the checklists for correlates and risk factor scores must be high. Thus, the studies that allow the most reliable conclusions to be drawn are prospective studies. To draw confident conclusions about causal risk factors, all three checklist scores must be high. Thus, in the absence of randomized clinical trials, confident conclusions can be drawn from studies with adequately controlled samples.

### 2.5. Statistic Analysis

The standard mean difference (SMD) with the 95% confidential interval (CI) was calculated for quantitative variables. The Cochran-Q and I^2^ statistics were used to evaluate the statistical heterogeneity. Specifically, if I^2^ resulted in being lower or equal to 50%, the variation in the studies was considered to be homogenous and the fixed effect model was adopted. If I^2^ was higher than 50%, there was significant heterogeneity between studies, and the random effects model was used. All *p*-values lower than 0.05 were considered statistically significant. The analysis was performed using RevMan software v. 5.4 (Cochrane Collaboration, Oxford, UK) and Comprehensive Meta-Analysis Software (Version 2) (Englewood, NJ, USA).

We undertook the sensitivity analysis with the exclusion method one study at a time. Therefore, the pooled effect size and corresponding CI were calculated after exclusion of one study at a time. A study that resulted in the inference changing after its exclusion was labeled a “sensitive study”.

We qualitatively analyzed the presence of publication bias from the asymmetry of the funnel plot, which suggested some missing studies on one side of the graph. Quantitative analysis of publication bias was performed using Egger’s intercept test, which assessed statistical significance of publication bias. In case of publication bias, unbiased estimates were calculated using the “trim and fill” method [25].

## 3. Results

**Figure 1:**
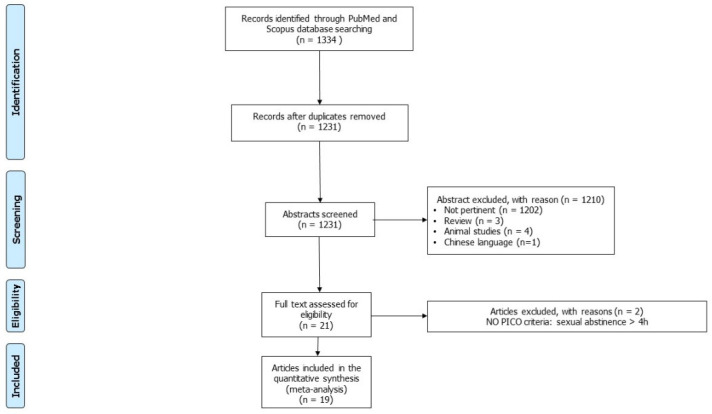
Flowchart of studies included.

The aforementioned search strategy identified 1334 records. After the exclusion of 103 duplicates, the remaining 1231 articles were screened. Of these, 1202 were judged to be not pertinent for their topic after reading their titles and abstracts, 3 were excluded because they were reviews, and 4 were excluded because they were studies conducted on animals. One study was excluded because it was written in Chinese. Twenty-one studies were carefully read. Among these, two studies were excluded for their experimental design. Finally, 19 articles met our inclusion criteria and were, therefore, included in the analysis [16,17,18,19,20,21,22,26,27,28,29,30,31,32,33,34,35,36,37]. All 19 articles evaluated the effects of a very short abstinence period on sperm parameters and among these, 5 articles also evaluated the SDF rate [16,21,31,32,36] (Figure 1). All studies were judged to be of low quality after the assessment with the Cambridge Quality Checklists (Table 2). The main characteristics of the studies included in the systematic review and meta-analysis are reported in Table 3.

### 3.1. Effects of a Short Period of Abstinence on Semen Parameters

#### 3.1.1. Semen Volume

##### Semen Volume: Qualitative Analysis

Eighteen studies evaluated the effect of a very short abstinence period on semen volume [16,17,18,19,20,21,22,26,27,28,29,30,31,32,33,34,36,37] (Table 3). Seventeen of them (94.4%) showed a lower semen volume after a very short sexual abstinence period [16,17,18,19,20,21,22,26,27,28,29,30,31,32,34,36,37]. Only one study did not find any significant change in semen volume in three healthy men after a very short abstinence period [32]. In detail, the authors evaluated the effects of four repeated ejaculations on the same day at two-hour intervals on semen parameters. Data of only the first two ejaculations (after 2 and 4 h, respectively) met our inclusion criteria and, therefore, they were included in our analysis. The authors showed a decreasing trend in semen volume in the second, third, and fourth collections after two hours of abstinence compared to the first one after 3–4 days of abstinence [32]. The very small sample size (*n* = 3) of this study may explain the lack of a statistically significant decrease in semen volume with shorter abstinence.

##### Semen Volume: Quantitative Analysis

The quantitative analysis of semen volume was performed in data extracted from 16 studies [16,17,18,19,20,22,27,28,29,30,31,32,33,34,36,37]. Although reported a reduction in semen volume, the study conducted by Shen et al. [21] was excluded from the quantitative analysis because no data regarding media, median, and standard deviation were reported in the article. The studies conducted by Tur-Kaspa et al. [27] and Alipour et al. [19,34] did not report data on median and standard deviation (SD) for semen volume, however, they were included in the quantitative analysis because they were calculated using the median, the minimum, and maximum values. Zverina et al. did not report if the men included in their study were normozoospermics or had OAT [26]. Furthermore, it was not possible to obtain these data from sperm parameters of the first semen collection because the authors did not report which WHO manual was used to perform semen analysis. For this reason, we decided to include the study conducted by Zverina et al. only in qualitative analysis but not in the quantitative one. The study by Hussein and colleagues [31] was considered twice since they evaluated the effects of a short period of abstinence on semen volume in a group of patients with oligo and/or asthenozoospermia and in a control group of fertile men. Mayorga-Torres and colleagues evaluated the effects of four repeated ejaculations on the same day at two-hour intervals (2, 4, 6, and 8 h) [32]. Therefore, Mayorga-Torres’s study was considered twice because we included the data after 2 h and 4 h and we excluded data regarding the samples after 6 and 8 h [32]. Again, Manna’s study [16] was also considered twice because it included a group of normozoospermic men and a group of patients with OAT. Furthermore, Tur-Kaspa and colleagues included two different groups: 27 patients with oligozoospermia and 23 OAT patients; therefore, this study was considered twice [27].

**Figure 2:**
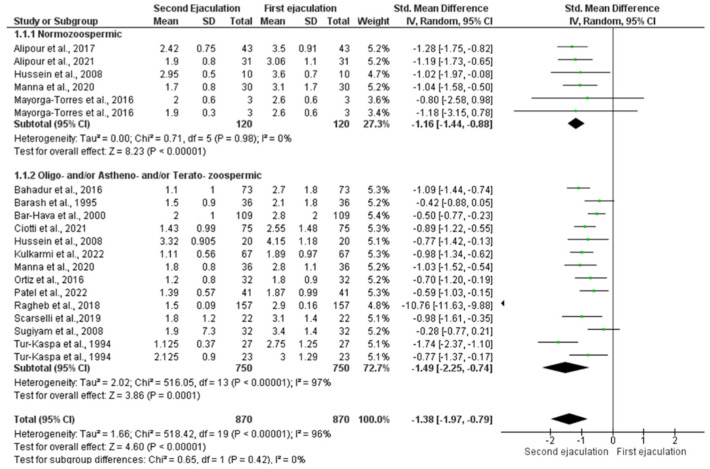
Forest plot of studies that evaluated the effects of a very short abstinence period on semen volume (expressed in mL). The following studies were included in the quantitative analysis (in order of appearance in the manuscript): Manna et al., 2020 [16], Bahadur et al., 2016 [17], Ortiz et al., 2016 [18], Alipour et al., 2017 [19], Ragheb et al., 2018 [20], Ciotti et al., 2021 [22], Tur-Kaspa et al., 1994 [27], Barash et al., 1995 [28], Bar-Hava et al., 2000 [29], Sugyam et al., 2008 [30], Hussein et al., 2008 [31], Mayorga-Torres et al., 2016 [32], Scarselli et al., 2019 [33], Alipour et al., 2021 [34], Kulkarmi et al., 2022 [36], and Patel et al., 2022 [37].

The statistical analysis showed a significant reduction in semen volume after a short period of abstinence in both normozoospermic men [SMD −1.16 (−1.44, −0.88); *p* < 0.00001] and oligozoospermic, asthenozoospermic, and/or teratozoospermic patients [SMD −1.49 (−2.25, −0.74); *p* = 0.0001] (Figure 2).

For the analysis of normozoospermic men, no inter-study heterogeneity was found, as demonstrated by the Q-test (Q-value = 0.71; *p*-value = 0.98) and I^2^ = 0%. Egger’s regression model and funnel plots reported no risk of bias (intercept = 0.096, 95% CI −0.96–1.16, *p* = 0.40) (Supplementary Figure S1A). At the sensitivity analysis, no study was sensitive enough to alter the above-reported results (Supplementary Figure S1B).

The analysis of patients with oligozoospermia, asthenozoospermia, and/or teratozoospermia showed the presence of inter-study heterogeneity (Q-value = 516.05; *p*-value = 0.000; I^2^ = 97%) and, therefore, the random model was used. Egger’s regression model and funnel plots reported risk of bias (intercept = −10.50, 95% CI −21.66–0.66, *p* = 0.03) (Supplementary Figure S2A). Three studies were the source of bias [20,27,29]. Once the data from these studies were excluded, heterogeneity decreased (Chi^2^ = 21.91, I^2^ = 30%) and the reduction in semen volume in the second sample remained significantly lower [SMD −0.79 (−0.96, −0.62); *p* < 0.00001]. However, at the sensitivity analysis, no study was sensitive enough to alter the above-reported results (Supplementary Figure S2B).

#### 3.1.2. Sperm Concentration

##### Sperm Concentration: Qualitative Analysis

Eighteen studies evaluated the effects of a short abstinence period on sperm concentration (Table 3) [16,17,18,19,20,21,22,26,27,28,29,30,31,32,33,34,35,36]. Among these, six studies described an increase in sperm concentration in the second ejaculation after a very short abstinence period [18,20,21,27,29,36]. Five of the six studies which demonstrated an improvement in sperm concentration were conducted in patients with oligozoospermia, asthenozoospermia, and/or teratozoospermia, whereas one study was conducted on 167 couples who underwent their first round of IVF but information on semen parameters of the first ejaculate of male partners was not reported [21]. Among these six studies, Tur-Kaspa et al. [27] evaluated two different groups: 27 patients with oligozoospermia and 23 with OAT. The authors found a statistically significant increase in sperm concentration only in patients with OAT, whereas patients with oligozoospermia had higher but not significant sperm concentration in the second ejaculation. In contrast, four studies reported a reduction in sperm concentration in the second sample [16,19,31,34]. Interestingly, all these studies were conducted on normozoospermic patients. In particular, Hussein et al. [31] included 20 patients with altered sperm parameters and 10 normozoospermic men. They found a statistically significant reduction in sperm concentration only in the second group. Likewise, Manna et al. [16] reported a decrease in sperm concentration only in 30 normozoospermic men and not in patients with OAT. Alipour et al. found a statistically significant reduction in sperm concentration in the second ejaculate collected after 2 h of the first one in 31 normozoospermic men [34]. The remaining studies did not find statistically significant changes in sperm concentration after a short period of abstinence

##### Sperm Concentration: Quantitative Analysis

Data from 16 studies were included in the quantitative analysis for the evaluation of the impact of a short abstinence period on sperm concentration [16,17,18,19,20,22,27,28,29,30,31,32,33,34,35,36]. The study by Shen et al. [24] was excluded from the quantitative analysis because no data on mean, median, or standard deviation were reported. For the same reasons reported in the paragraph on semen volume, the studies conducted by Tur-Kaspa et al. [27], Hussein et al. [31], Mayorga-Torres et al. [32], and Manna et al. [16] were considered twice in the quantitative analysis. The studies conducted by Tur-Kaspa [27] and Alipour et al. in 2017 and 2021 [19,34] did not report data of mean and standard deviation for sperm concentration but they were included in the quantitative analysis because media ± SD were calculated using the median, the minimum, and maximum values. As reported for semen volume, the study conducted by Zverina et al. [26] was not included in the quantitative analysis because authors did not report if men included in their study were normozoospermic or OAT.

**Figure 3:**
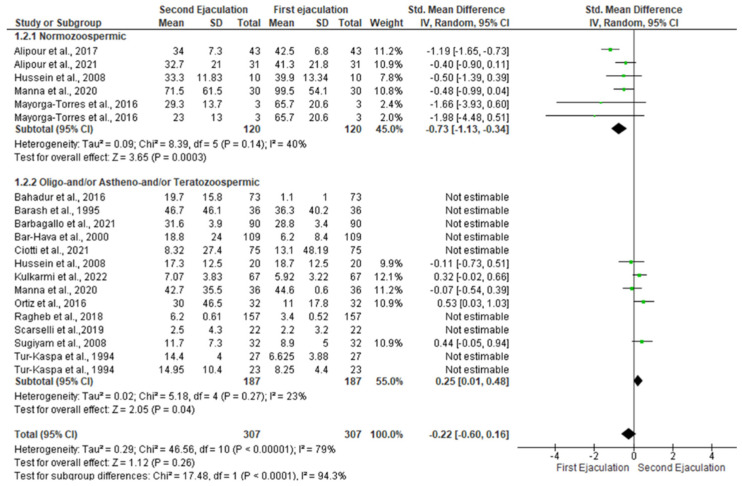
Forest plot of studies that evaluated the effects of a very short abstinence period on sperm concentration (expressed in mil/mL). The following studies were included in the quantitative analysis (in order of appearance in the manuscript): Manna et al., 2020 [16], Bahadur et al., 2016 [17], Ortiz et al., 2016 [18], Alipour et al., 2017 [19], Ragheb et al., 2018 [20], Ciotti et al., 2021 [22], Tur-Kaspa et al., 1994 [27], Barash et al., 1995 [28], Bar-Hava et al., 2000 [29], Sugyam et al., 2008 [30], Hussein et al., 2008 [31], Mayorga-Torres et al., 2016 [32], Scarselli et al., 2019 [33], Alipour et al., 2021 [34], Barbagallo et al.,2021 [35], and Kulkarmi et al., 2022 [36].

The statistical analysis showed a reduction in sperm concentration in the second ejaculation of normozoospermic men [SMD −0.73 (−1.13, −0.34); *p* = 00003]. In contrast, sperm concentration significantly increased in patients with abnormal sperm parameters [SMD 0.87 (0.22, 1.51); *p* = 0.009] (Figure 3).

The I^2^ (40%) revealed no inter-study heterogeneity in the studies conducted on normozoospermic men. However, this was not confirmed by the Q-test (Q-value = 11.141; *p*-value = 0.049). Egger’s regression model and funnel plots reported no risk of bias (intercept = −1.38, 95% CI −5.03–2.27, *p* = 0.18) (Supplementary Figure S3A). At the sensitivity analysis, no study was sensitive enough to alter the above-reported results (Supplementary Figure S3B). The analysis of patients with abnormal sperm parameters revealed the presence of inter-study heterogeneity, as confirmed by the Q-test (Q-value = 440.766; *p*-value = 0.000) and I^2^ = 97%. The analysis of publication bias revealed no source of biases at Egger’s regression model and funnel plots (intercept = 2.06, 95% CI −10.91–15.03, *p* = 0.36) (Supplementary Figure S4A). At the sensitivity analysis, no study was sensitive enough to alter the above-reported results (Supplementary Figure S4B).

#### 3.1.3. Total Sperm Motility

##### Total Sperm Motility: Qualitative Analysis

Fifteen studies evaluated the impact of a very short abstinence period on total sperm motility [16,18,19,20,22,26,27,28,29,30,32,33,34,35,36] (Table 3). The study by Hussein et al. [31] was excluded because they did not report total sperm motility but only partial data regarding spermatozoa with rapid progressive (A), slow progressive (B), and non-progressive motility (C), and non-motile (D) spermatozoa. The authors described a significant increase in A and B and a significant decrease in C. The study by Bahadur et al. was excluded for the same reason [17]. They described a significant increase in A and a significant decrease in B, C, and D. Similarly, Shen et al. reported a significant improvement in motile sperm count in the second ejaculation compared to the first one [21]. Ten of the fifteen studies (66.6%) demonstrated a statistically significant increase in total sperm motility in the second ejaculate [16,18,20,22,28,29,30,35,36]. In particular, 10 out of 11 studies were conducted on patients with altered sperm parameters and only the study of Alipour et al. reported an increase in total sperm motility in 43 normozoospermic men [19]. Manna et al. [16] included 30 normozoospermic men and 36 OAT patients, although, in both groups, they found an increase in total sperm motility, the improvement reached statistical significance only in OAT patients. Four of the sixteen studies were unable to show significant alterations in sperm motility in the consecutive ejaculate collected within 4 h [27,32,33,34]. Only one study reported a statistically significant reduction in sperm motility [26]. However, this study was performed in 1988, and the WHO manual was not used to perform the semen analysis. The authors evaluated sperm motility and sperm velocity of the second ejaculation collected 1 h after the first one, in 107 men with an infertile marriage for at least one year. The different methodologies used could explain the different results from all the others.

##### Total Sperm Motility: Quantitative Analysis

**Figure 4:**
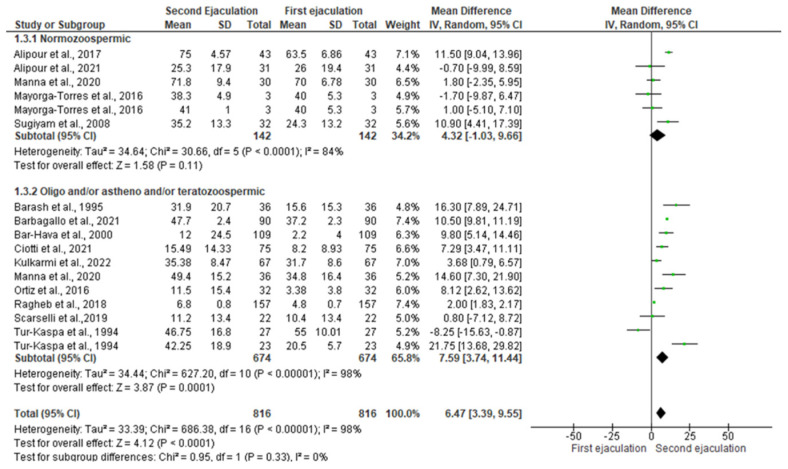
Forest plot of studies that evaluated the effects of a very short abstinence period on total sperm motility (expressed in percentage). The following studies were included in the quantitative analysis (in order of appearance in the manuscript): Manna et al., 2020 [16], Ortiz et al., 2016 [18], Alipour et al., 2017 [19], Ragheb et al., 2018 [20], Ciotti et al., 2021 [22], Tur-Kaspa et al., 1994 [27], Barash et al., 1995 [28], Bar-Hava et al., 2000 [29], Sugyam et al., 2008 [30], Mayorga-Torres et al., 2016 [32], Scarselli et al., 2019 [33], Alipour et al., 2021 [34], Barbagallo et al., 2021 [35], and Kulkarmi et al., 2022 [36].

The data on the effects of a short abstinence period on total sperm motility could be extracted from 14 studies [16,18,19,20,22,27,28,29,30,32,33,34,35,36]. The study conducted by Zverina et al. was not included in the quantitative analysis because the authors did not report if the men included in their study were normozoospermic or had OAT [26]. As for other sperm parameters, the studies conducted by Tur-Kaspa et al. [27], Mayorga-Torres et al. [32], Manna et al. [16] were considered twice in the quantitative analysis. For the studies conducted by Tur-Kaspa [27] et al. and Alipour et al. 2017 and 2022 [19,34], the mean of total sperm motility was calculated using the median, the minimum, and maximum values. The statistical analysis showed that a second ejaculation after a short period of abstinence improved total sperm motility only in patients with abnormal sperm parameters [SMD 7.59 (3.74, 11.44); *p* = 0.0001] without any significant changes in normozoospermic men [SMD 4.32 (−1.03, 9.66); *p* = 0.11] (Figure 4).

In the analysis of normozoospermic men, inter-study heterogeneity was observed, as confirmed by the Q-test (Q-value = 37.389; *p*-value = 0.000) and the I^2^ = 84%. Therefore, the random model was used. Egger’s regression model and funnel plots reported no risk of bias (intercept = −1.207, 95% CI −10.03–7.61, *p* = 0.36) (Supplementary Figure S5A). At the sensitivity analysis, no study was sensitive enough to alter these results (Supplementary Figure S5B).

The analysis of subgroups with oligozoospermia, asthenozoospermia, and/or teratozoospermia (Chi^2^ = 627.2, I^2^ = 98%) found significant inter-study heterogeneity (Q-value = 340.336; *p*-value = 0.000; I^2^ = 98%). At Egger’s regression model and funnel plots, no risk of bias was found (intercept = 0.467, 95% CI −14.14–15.08, *p* = 0.47) (Supplementary Figure S6A). Furthermore, no study was sensitive enough to alter the above-mentioned results (Supplementary Figure S6B).

#### 3.1.4. Progressive Sperm Motility

##### Progressive Sperm Motility: Qualitative Analysis

Ten studies evaluated the effects of a very short abstinence period on sperm progressive motility [16,18,20,22,29,32,34,35,36,37] (Table 3). The studies by Hussein et al. [31] and Bahadur et al. [17] were excluded because they did not report the value of progressive sperm motility (A + B) but only partial data regarding A, B, C, and D. However, Hussein et al. described a significant increase in A and B, whereas Bahadur et al. reported a significant increase in A and a significant decrease in B. Nine of the ten studies included described an increase in sperm progressive motility in the second ejaculate. Only two studies did not show any statistically significant change in the second ejaculate for progressive sperm motility [32,37]. In particular, the study conducted by Mayorga-Torres et al. [32] was conducted in three normozoospermic patients. Therefore, the small sample size of this study may explain the different results compared to other studies. Furthermore, most of the studies that showed an improvement of progressive sperm motility included patients with oligozoospermia, asthenozoospermia, and/or teratozoospermia. In particular, Manna et al., [16] included both normozoospermic and OAT patients, however, they found a statistically significant increase in progressive sperm motility only in the OAT group.

##### Progressive Sperm Motility: Quantitative Analysis

Data from ten studies were included in the quantitative analysis to evaluate the impact of a very short abstinence period on progressive sperm motility [16,18,20,22,29,32,34,35,36,37]. As for other parameters, the study by Shen et al. was not included in the quantitative analysis because no data on mean, median, or ST were reported [21]. The studies conducted by Mayorga-Torres et al. [32] and Manna et al. [16] were considered twice in the quantitative analysis for the same reasons reported previously.

**Figure 5:**
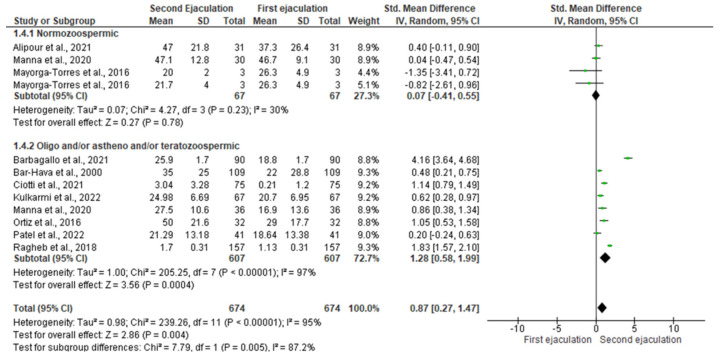
Forest plot of studies that evaluated the effects of a very short abstinence period on progressive sperm motility (expressed as a percentage). The following studies were included in the quantitative analysis (in order of appearance in the manuscript): Manna et al., 2020 [16], Ortiz et al., 2016 [18], Ragheb et al., 2018 [20], Ciotti et al., 2021 [22], Bar-Hava et al., 2000 [29], Mayorga-Torres et al., 2016 [32], Alipour et al., 2021 [34], Barbagallo et al., 2021 [35], Kulkarmi et al., 2022 [36], and Patel et al., 2022 [37].

As for total sperm motility, the statistical analysis showed that a second ejaculation after a very short abstinence period improved progressive sperm motility only in patients with abnormal sperm parameters [SMD 1.28 (0.58, 1.99); *p* = 0.0004] without any significant changes in normozoospermic men [SMD −1.55 (−6.96, 3.85); *p* = 0.57] (Figure 5).

The analysis of normozoospermic men found the presence of inter-study heterogeneity at the Q-test (Q-value = 6.645; *p*-value = 0.084), but not at the I^2^ (30%). Egger’s regression model and funnel plots showed the presence of risk of bias (intercept = −2.41, 95% CI −5.70–0.87, *p* = 0.04) (Supplementary Figure S7A). However, no study was sensitive enough to alter the above-mentioned results (Supplementary Figure S7B).

Furthermore, inter-study heterogeneity was found for the subgroups of oligozoospermic, asthenozoospermic, and/or teratozoospermic patients (Q-value = 207.899; *p*-value = 0.000; I^2^ = 97%). Egger’s regression model and funnel plots reported no risk of bias (intercept = 4.364, 95% CI −14.98–23.71, *p* = 0.30) (Supplementary Figure S8A). At the sensitivity analysis, no study was sensitive enough to alter the above-reported results (Supplementary Figure S8B).

#### 3.1.5. Sperm Morphology

##### Sperm Morphology: Qualitative Analysis

Ten studies investigated the impact of a very short abstinence period on sperm morphology [16,17,20,21,22,26,28,33,35,37] (Table 3). Five studies showed no statistically significant changes in sperm morphology in the second ejaculate [21,26,28,33,35]. The remaining five studies demonstrated that a very short abstinence period improved sperm morphology. In particular, Manna et al. [16] reported a statistically significant improvement of sperm morphology in the second ejaculation only in OAT patients and not in normozoospermic men. Almost all (four out of five) participants reported an improvement of sperm morphology after a very short abstinence period in a study conducted in patients with abnormal sperm parameters, whereas only one was conducted in normozoospermic men [21].

##### Sperm Morphology: Quantitative Analysis

**Figure 6:**
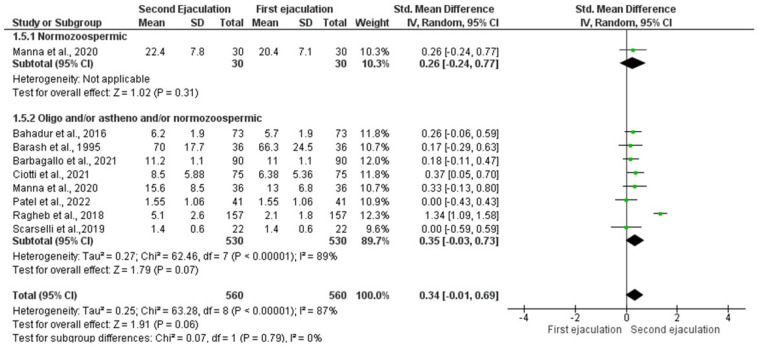
Forest plot of studies that evaluated the effects of a very short abstinence period on sperm morphology (expressed as a percentage). The following studies were included in the quantitative analysis (in order of appearance in the manuscript): Manna et al., 2020 [16], Bahadur et al., 2016 [17], Ragheb et al., 2018 [20], Ciotti et al., 2021 [22], Barash et al., 1995 [28], Sarselli et al., 2019 [33], Barbagallo et al., 2021 [35].

Quantitative analysis on sperm morphology was evaluated on data from eight studies [16,17,20,22,28,33,35]. The study by Manna et al. [16] was considered twice because they included a group of normozoospermic men and a group of patients with OAT. The study by Shen et al. [21] was not included in the quantitative analysis because no data on mean, median, or standard deviation were reported. As for other sperm parameters, the study by Zverina et al. [26] was not included in the quantitative analysis because the authors did not report if the men included in their study were normozoospermics or OAT. In particular, the statistical analysis did not show a significant improvement in sperm morphology in patients with abnormal sperm parameters [SMD 0.35 (−0.03, 0.73); *p* = 0.07] (Figure 6).

Only one study [25] included a subgroup of normozoospermic men. Therefore, analysis of publication bias and sensitivity analysis could not be performed in this sub-group. Inter-study heterogeneity was found for the subgroups of oligozoospermic, asthenozoospermic, and/or teratozoospermic patients (Q-value = 62.76; *p*-value = 0.000; I^2^ = 89%). Egger’s regression model and funnel plots reported the presence of risk of bias (intercept = −7.10, 95% CI −15.06–0.85, *p* = 0.03) (Supplementary Figure S9A). However, at the sensitivity analysis, no study was sensitive enough to alter the above-reported results (Supplementary Figure S9B).

#### 3.1.6. Sperm DNA Fragmentation

##### Sperm DNA Fragmentation: Qualitative Analysis

Five studies investigated the effect of a very short abstinence period on the SDF rate [16,21,31,32,36] (Table 3). Four of the five studies reported a statistically significant reduction in the SDF rate after a very short abstinence period. In particular, Hussein et al. [31] found a statistically significant reduction in spermatozoa with severe DNA damage in the second ejaculation in both patients with abnormal sperm parameters and control men. In the study, SDF was evaluated using the Comet assay. Shen et al. reported a statistically significant reduction in SDF rate using the Sperm Chromatin Structure Assay (SCSA) in 167 patients; 61.1% of the patients enrolled in their study had normal sperm parameters [21]. According to Shen et al., Manna and colleagues reported a statistically significant reduction in SDF rate in both normozoospermic men and OAT patients using the Halosperm kit [16]. Furthermore, Kulkarmi et al. also showed lower SDF rates evaluated using the Qwik Check DFI test assay in conventional bright-field microscopy, in the second ejaculates compared to the first one of 67 oligozoospermic patients [36]. Only one study conducted by Torres et al. did not find any statistically significant change in the DNA fragmentation index evaluated using the SCSA kit. The small sample size (*n* = 3) could explain why this study did not find any change in the SDF rate. Indeed, the authors investigated SDF in only three healthy men at the second, third, and fourth evaluations after two hours of abstinence in comparison to the first evaluation after 2–4 days of abstinence [32].

##### Sperm DNA Fragmentation: Quantitative Analysis

Quantitative analysis of the SDF rate was evaluated in four studies [16,31,32,36]. The study by Shen et al. was not included in the quantitative analysis because no data on mean, median, or standard deviation was reported [21]. For the same reasons reported for other sperm parameters, the studies conducted by Torres et al. [32] and Manna et al. [16] were considered twice for quantitative analysis.

**Figure 7:**
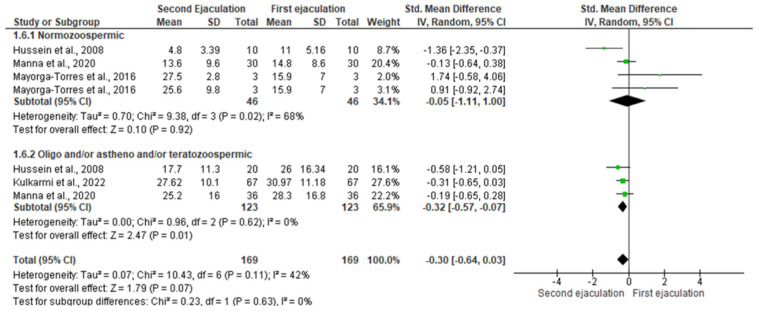
Forest plot of studies that evaluated the effects of a very short abstinence period on sperm DNA fragmentation (expressed as a percentage). The following studies were included in the quantitative analysis (in order of appearance in the manuscript): Manna et al., 2020 [16], Hussein et al., 2008 [31], Mayorga-Torres et al., 2016 [32], Kulkarmi et al., 2022 [36].

In particular, the statistical analysis showed that a second ejaculation after a very short abstinence period improved the SDF rate only in patients with abnormal sperm parameters [SMD −3.92 (−6.97, −0.87); *p* = 0.01] without any significant changes in normozoospermic men [SMD −2 (−4.72, 0.73); *p* = 0.15] (Figure 7).

In the group of normozoospermic men, the analysis showed the presence of inter-study heterogeneity (Q-value = 13.648; *p*-value = 0.003; I^2^ = 68%). No risk of bias was found at Egger’s regression model and funnel plots (intercept = 1.610, 95% CI −8.507–11.727, *p* = 0.28) (Supplementary Figure S10A). No study was sensitive enough to alter the above-reported results (Supplementary Figure S10B).

In addition, no inter-study heterogeneity was found for the subgroups of oligozoospermic, asthenozoospermic, and/or teratozoospermic patients (Q-value = 1011; *p*-value = 0.603; I^2^ = 0%). No risk of bias was found at Egger’s regression model and funnel plots (intercept = −1.353, 95% CI −26.50–23.79, *p* = 0.309) (Supplementary Figure S11A). The study by Kulkarmi et al., 2022 [29] was sensitive enough to change these results. Indeed, its removal led to the loss of significance (Supplementary Figure S11B).

## 4. Discussion

The optimal period of sexual abstinence is still a matter of debate. Data on the effect of abstinence length on semen parameters are extremely heterogeneous and many publications from several decades ago are not yet conclusive. A systematic review conducted by Hanson and colleagues including 28 studies investigated the impact of abstinence on semen parameters and fertility outcome. Analysis of publications showed that a longer abstinence was associated with increases in semen volume and sperm count. On the contrary, studies evaluating the effect of abstinence on motility, morphology, and SDF rate, although contradictory and not conclusive, showed a trend toward improvements with shorter abstinence period [15]. However, the authors did not establish any cut off to distinguish short and long abstinence period in their inclusion criteria.

Over time, many authors have supported the potential improvement of semen parameters in a second ejaculation collected after a very short period (within a few hours) from the first semen collection. However, data are still controversial. To our knowledge, this systematic review and meta-analysis, for the first time, pooled evidence for the influence of a very short abstinence period on sperm parameters and the SDF rate.

Our quantitative analysis showed a significant reduction in sperm volume after a very short abstinence period in both normozoospermic men and OAT patients. Sperm volume reflects the secretory activity of the accessory glands and the subsequent smooth muscle contractions that empty each gland in response to autonomous nerve stimulation elicited by sexual arousal [10]. Previous studies did not find consistent relationship between semen volume and fertility [14,38]. Of course, a minimal volume of semen is necessary for conception, and hypoposia should be investigated because it could reflect different pathological conditions (such as hypotestosteronemia, abnormalities of the neuroreceptor systems, retrograde ejaculation, and obstructive diseases) [9]. According to the last edition of the WHO manual for human sperm analysis, sperm volume should be equal to or more than 1.4 mL [10]. Sexual abstinence significantly influences sperm volume. Indeed, sperm volume was shown to increase by 11.9% per day in the first 4 days following ejaculation [39].

The present analysis also showed a decrease in sperm concentration in the second ejaculation of normozoospermic men and, conversely, a significant increase in patients with abnormal sperm parameters. Interestingly, also for total and progressive sperm motility, the improvement in the second ejaculation was statistically significant only in patients with OAT, without any changes in normozoospermic men. Furthermore, an improvement in sperm morphology was found in OAT patients but it did not reach statistical significance. Therefore, the results of the present meta-analysis support previous evidence on the beneficial effect of a very short abstinence period, especially in patients with abnormal sperm parameters.

The mechanism of sperm quality improvement in the second ejaculation collected after a short interval is unclear. Among the different reasons hypothesized, a different duration of the epididymal transit could play the main role. Human spermatozoa are produced in the seminiferous tubules and then stored in the epididymis. The length of spermatozoon epididymal transit time of spermatozoa ranges from 2 to 11 days [40] and its duration is possibly related to the rate of passage through the cauda which, in turn, can be influenced by the ejaculatory frequency [41]. During epididymal transit and storage, spermatozoa are exposed to high levels of reactive oxygen species (ROS). The hostile environment in the epididymis may be related to several causes, including dysfunctions or partial obstructions of the epididymis itself, stasis of seminal fluids, accumulation of senescent-degenerating spermatozoa, and packing of cells involved in the removal of aging spermatozoa [42,43]. Therefore, a short period of abstinence could decrease the time of exposure of spermatozoa to the harmful effects of ROS in the cauda epididymis and, in turn, may result in a “healthier” population of spermatozoa [13]. Previous studies have reported that prolonged exposure to ROS arising from dead spermatozoa and leukocytes may be one reason for the association between reduction in sperm quality and an increase in SDF rate with low ejaculation frequencies [44]. According to this hypothesis, Shen and colleagues found an increased total antioxidant capacity in ejaculates from short (1–3 h) compared with long (3–7 days) length of abstinence [21]. Moreover, Torres and colleagues found a decreasing trend of intracellular ROS production in four repeated ejaculations on the same day at two-hour intervals, and the difference became statistically significant at the fourth evaluation in comparison to the first one [32].

Interestingly, Johnson and Varner reported that the duration of epididymal transit was three times longer in patients with oligozoospermia than in men with normozoospermia [45]. Therefore, spermatozoa of patients with severe OA are stationed in the genital tract for a prolonged time and, in turn, are more damaged by oxidative stress. This might explain the greater improvement of sperm quality after a very short period of abstinence in patients with abnormal sperm parameters compared to normozoospermic men.

Furthermore, during the epididymal transit, several epigenetic modifications occur [46] and it is a fundamental step for spermatogenesis, sperm maturation, and the fertilization process [47]. In 2019, Shen and colleagues confirmed the potential molecular diversity of spermatozoa ejaculated after 1–3 h compared to 3–7 days by proteomic techniques [21]. Interestingly, the main differences were found in the expression of proteins highly involved in sperm motility and capacitation. The acrosome reaction capability of spermatozoa was markedly elevated after 1–3 h of abstinence [21]. To date, the role of these proteins in abstinence-related sperm function is still unclear. However, these findings suggest that a short abstinence period can alter the expression of sperm proteins, which may be one of the reasons why short sexual abstinence may improve sperm quality [48].

The investigation of biofunctional sperm parameters after a very short abstinence period could help us to understand the origin of this improvement. As reported in our results, a very short abstinence period is associated with a significant improvement in the SDF rate. This can be caused by extrinsic factors or intrinsic factors, including increased oxidative stress [49]. Therefore, the reduction in sperm SDF supports previous hypotheses that a short period of abstinence could decrease the time of exposure of spermatozoa to high levels of ROS. Few studies have investigated the effects of a very short sexual abstinence on other biofunctional sperm parameters. Shen and coworkers found a higher sperm mitochondrial membrane potential (MMP) in ejaculates from short (1–3 h) compared to long (3–7 days) length of abstinence [21]. MMP is a marker of sperm mitochondrial function that strictly correlates with sperm motility [50]. Mayorga-Torres and colleagues demonstrated that sperm MMP and plasma membrane integrity remained stable throughout four repeated ejaculations on the same day at two-hour intervals [32]. Scarselli et al. found an increase in the percentage of mature chromatin in ejaculates obtained after a very short abstinence time (1 h) [33]. Sperm chromatin structure could be important for the maintenance of the right epigenetic patterns during spermatogenesis. Epigenetic events in OAT patients directly influence embryogenesis. It is known that failure of ART treatment in couples with male partner infertility could be related to epigenetic alterations of blastocysts [47].

Furthermore, it was also speculated that the changes in seminal plasma composition after a very short period of abstinence might influence sperm quality. In particular, Alipour and colleagues compared the seminal plasma metabolomics profile of two consecutive ejaculates collected from normozoospermic men. The first sample was collected after an abstinence period of 4–7 days, whereas the second one was collected after a very short (2 h) abstinence period. The authors found a lower absolute amount of all metabolites in the second ejaculate [34]. This may be related to the insufficient time available for the secretion and accumulation of these metabolites by accessory sex glands, including the epididymis. However, the contemporary lower number of spermatozoa in the second ejaculate resulted in increased absolute amounts of pyruvate and taurine per spermatozoa, together with an improvement of sperm motility in these samples. Therefore, the authors speculated that changes in the seminal plasma composition might influence spermatozoa motility and kinematic parameters [34].

All the studies included in this systematic review and meta-analysis were judged as of fair quality at the quality analysis. Nevertheless, some limitations should be considered. The main limitation is that many of the included studies were observational. Furthermore, the analysis revealed a large heterogeneity in the studies included. This inter-study heterogeneity could be partly explained by the different methods to evaluate semen parameters and the SDF rate (Table 3). Many of the studies included have a relatively small sample size. The significance of data from smaller studies should not be ignored, although the larger studies included in our analysis had the statistical power to provide more convincing evidence. Another limitation is that the majority of publications included in the present meta-analysis evaluated different sperm parameters but not all studies evaluated the same parameters, making it difficult to draw a strong conclusion about some of these. Furthermore, cigarette smoking, caffeine intake, and lifestyle were not analyzed, although a significant relationship between the aforementioned factors with sperm quality was concluded by previous studies [4,51,52]. Further prospective randomized and larger studies are needed to evaluate the effects of a short abstinence period on sperm quality. Further studies should evaluate the effects of a very short abstinence period on biofunctional sperm parameters to better understand the reason for the improvement in sperm quality. Furthermore, future studies should evaluate the sperm parameters of the second ejaculation also based on the sexual abstinence length before the first collection.

## 5. Conclusions

This is the first systematic review and meta-analysis, which investigate the impact of a very short abstinence period on sperm parameters and the SDF rate. Our results suggest that a second ejaculation collected after a very short period from the first one contains spermatozoa of better quality, in terms of sperm concentration, total and progressive motility, and the SDF rate in patients with abnormal sperm parameters.

These results could have important implications in both natural and assisted reproductive technologies. For couples in reproductive age, these data suggest that more frequent intercourse with a very short sexual abstinence period could enhance conception.

## Figures and Tables

**Table 1:** Selection criteria in included studies (PICOS) (Population, Intervention, Comparison/Comparator, Outcomes, Study design) model of the current systematic review and meta-analysis.

	Inclusion	Exclusion
Population	Men of reproductive age	Azoospermia, age < 18 years
Intervention	Short-second ejaculation (within 4 h)	Second ejaculation > 4 h
Comparison	Ejaculation after an abstinence sexual period between 2–7 days	/
Outcome	Sperm conventional parameters (semen volume, sperm concentration, sperm progressive motility, sperm total motility, sperm morphology) and SDF	/
Study type	Observational, cohort, cross-sectional, and case–control	Case reports, comments, letters to the editor, systematic or narrative reviews, in vitro studies, studies on animals

Abbreviations: SDF, sperm DNA fragmentation.

**Table 2:** Evaluation of study quality using “The Cambridge Quality Checklists”.

Authors and Year of Publication	Checklist for Correlates	Checklist for Risk Factors	Checklist for Casual Risk Factors	Total
Zverina et al., 1988 [26]	1	1	3	5/15
Tur-Kaspa et al., 1994 [27]	1	1	3	5/15
Barash et al., 1995 [28]	1	1	3	5/15
Bar-Hava et al., 2000 [29]	1	1	3	5/15
Sugiyam et al., 2008 [30]	1	1	3	5/15
Hussein et al., 2008 [31]	1	1	3	5/15
Bahadur et al., 2016 [17]	1	1	3	5/15
Ortiz et al., 2016 [18]	1	1	3	5/15
Mayorga-Torres et al., 2016 [32]	2	1	3	6/15
Alipour et al., 2017 [19]	2	1	3	6/15
Ragheb et al., 2018 [20]	1	1	3	5/15
Shen et al., 2019 [21]	2	1	3	6/15
Scarselli et al., 2019 [33]	2	1	3	6/15
Manna et al., 2020 [16]	2	1	3	6/15
Ciotti et al., 2021 [22]	1	1	3	5/15
Alipour et al., 2021 [34]	2	1	3	6/15
Barbagallo et al., 2021 [35]	2	1	3	6/15
Kulkarmi et al., 2022 [36]	1	1	3	6/15
Patel et al., 2022 [37]	1	1	3	5/15

**Table 3:** Effects of a very short abstinence period (within 4 h) on sperm conventional parameters and DNA fragmentation rate.

Authors and Years	Patients	Abstinence Period of First Ejaculate	Abstinence Period of Second Ejaculate	Method Used for Semen Analysis	Semen Parameters					Method of Evaluation of SDF	SDF
					Volume	Concentration	Total Motility	Progressive Motility	Normal Morphology		
Zverina et al., 1988 [26]	107 partners of infertile couples	3–6 days	60 min	NA	↓	-	↓	NA	-	NA	NA
Tur-Kaspa et al., 1994 [27]	27 Oligo	3 days	<4 h	NA	↓	-	-	NA	NA	NA	NA
	23 OAT	3 days	<4 h	NA	↓	↑	-	NA	NA	NA	NA
Barash et al., 1995 [28]	36 OAT	3 days	2 h	NA	↓	-	↑	NA	-	NA	NA
Bar-Hava et al., 2000 [29]	109 severe OAT	NA	1 h	NA	↓	↑	↑	↑	NA	NA	NA
Sugiyam et al., 2008 [30]	32 OAT	3–5 days	30–60 min	NA	↓	-	↑	NA	NA	NA	NA
Hussein et al., 2008 [31]	20 Oligo or OAT	3 days	<1–3 h	WHO, 1999	↓	-	NA	NA	NA	Comet assay	↓
	10 Normo	3 days			-	↓	NA	NA	NA		↓
Bahadur et al., 2016 [17]	73 Oligo	2–7 days	40 min	WHO, 2010	↓	-	NA	NA	↑	NA	NA
Ortiz et al., 2016 [18]	32 OA	1–5 days	Less 1 h	WHO, 2010	↓	↑	↑	↑	NA	NA	NA
Mayorga-Torres et al., 2016 [32]	3 Normo	3–4 days	2 h	WHO, 2010	-	-	-	-	NA	SCSA	-
			4 h		-	-	-	-	NA		-
Alipour et al., 2017 [19]	43 Normo	At least 4 days	2 h	Sperm Class Analyzer CASA system	↓	↓	↑	NA	NA	NA	NA
Ragheb et al., 2018 [20]	157 OAT	3–7 days	1–3 h	WHO, 2010	↓	↑	↑	↑	↑	NA	NA
Shen et al., 2019 [21]	167 Normo	3–7 days	1–3 h	WHO, 2010	↓	↑	NA	NA	-	SCSA	-
	20 Normo	3–7 days	1–3 h	WHO, 2010	-	-	NA	NA	↑	SCSA	↓
Scarselli et al., 2019 [33]	22 OAT	2–5 days	1 h	WHO, 2010	↓	-	-	NA	-	-	NA
Manna et al., 2020 [16]	30 Normo	2–7 days	1 h	WHO, 2010	↓	↓	-	-	-	SCD test (Halosperm kit)	↓
	36 OAT	2–7 days	1 h	WHO, 2010	↓	-	↑	↑	↑	SCD test (Halosperm kit)	↓
Ciotti et al., 2021 [22]	75 Severe OAT	2–3 days	2 h	NA	↓	-	↑	↑	↑	NA	NA
Alipour et al., 2021 [34]	31 Normo	4–7 days	2 h	WHO, 2010; Sperm Class Analyzer	↓	↓	-	↑	NA	NA	NA
Barbagallo et al., 2021 [35]	90 Severe OA	2–7 days	1 h	WHO, 2010	NA	-	↑	↑	-	NA	NA
Kulkarmi et al., 2022 [36]	67 Oligo	2–7 days	1–3 h	WHO, 2010	↓	↑	↑	↑	NA	SCD test (Qwik Check DFI test assay)	↓
Patel et al., 2022 [37]	41 Severe OAT	2–7 days	1 h	WHO,2010	↓	NA	NA	-	-	NA	NA

Abbreviations: ↓ = reduction; ↑ = increase; - = no significant changes; NA = not available; SDF = sperm DNA fragmentation; CASA = computer-assisted sperm analysis; DFI = DNA fragmentation index; NA = not available; normo = normozoospermia; oligo = oligozoospermia; OA = oligoasthenozoospermia; OAT = oligoasthenoteratozoospermia; WHO = World Health Organization; SCD = sperm chromatin dispersion; SCSA = Sperm chromatin structure assay.

## Data Availability

The data are available upon request from the corresponding author.

## Supplementary Materials

The following supporting information can be downloaded at: https://www.mdpi.com/article/10.3390/jcm11247303/s1, Table S1: PRISMA checklist; Figure S1: Funnel plot (A) and sensitivity analysis (B) of studies that evaluated the effects of a very short abstinence period on seminal fluid volume in normozoospermic men; Figure S2: Funnel plot (A) and sensitivity analysis (B) of studies that evaluated the effects of a very short abstinence period on seminal fluid volume in oligo- and/or astheno- and/or teratozoospermic men; Figure S3: Funnel plot (A) and sensitivity analysis (B) of studies that evaluated the effects of a very short abstinence period on sperm concentration in normozoospermic men; Figure S4: Funnel plot (A) and sensitivity analysis (B) of studies that evaluated the effects of a very short abstinence period on sperm concentration in oligo- and/or astheno- and/or teratozoospermic men; Figure S5: Funnel plot (A) and sensitivity analysis (B) of studies that evaluated the effects of a very short abstinence period on sperm total motility in normozoospermic men; Figure S6: Funnel plot (A) and sensitivity analysis (B) of studies that evaluated the effects of a very short abstinence period on sperm total motility in oligo- and/or astheno- and/or teratozoospermic men; Figure S7: Funnel plot (A) and sensitivity analysis (B) of studies that evaluated the effects of a very short abstinence period on sperm progressive motility in normozoospermic men; Figure S8: Funnel plot (A) and sensitivity analysis (B) of studies that evaluated the effects of a very short abstinence period on sperm progressive motility in oligo- and/or astheno- and/or teratozoospermic men; Figure S9: Funnel plot (A) and sensitivity analysis (B) of studies that evaluated the effects of a very short abstinence period on sperm morphology in oligo- and/or astheno- and/or teratozoospermic men; Figure S10: Funnel plot (A) and sensitivity analysis (B) of studies that evaluated the effects of a very short abstinence period on sperm DNA fragmentation in normozoospermic men; Figure S11: Funnel plot (A) and sensitivity analysis (B) of studies that evaluated the effects of a very short abstinence period on sperm DNA fragmentation in oligo- and/or astheno- and/or teratozoospermic men. Reference [53] is cited in Supplementary Materials.
