# Supplementary material for: The Impact of a Very Short Abstinence Period on Conventional Sperm Parameters and Sperm DNA Fragmentation: A Systematic Review and Meta-Analysis
Source: J Clin Med. 2022 Dec 8;11(24):7303. doi: 10.3390/jcm11247303 (PMC9782170; doi:10.3390/jcm11247303)

Table S1. PRISMA 2009 Checklist

Section/topic	#	Checklist item	Reported on page #
**TITLE**			
Title	1	Identify the report as a systematic review, meta-analysis, or both.	1
**ABSTRACT**			
Structured summary	2	Provide a structured summary including, as applicable: background; objectives; data sources; study eligibility criteria, participants, and interventions; study appraisal and synthesis methods; results; limitations; conclusions and implications of key findings; systematic review registration number.	1
**INTRODUCTION**			
Rationale	3	Describe the rationale for the review in the context of what is already known.	1-2
Objectives	4	Provide an explicit statement of questions being addressed with reference to participants, interventions, comparisons, outcomes, and study design (PICOS).	3
**METHODS**			
Protocol and registration	5	Indicate if a review protocol exists, if and where it can be accessed (e.g., Web address), and, if available, provide registration information including registration number.	NA
Eligibility criteria	6	Specify study characteristics (e.g., PICOS, length of follow-up) and report characteristics (e.g., years considered, language, publication status) used as criteria for eligibility, giving rationale.	3
Information sources	7	Describe all information sources (e.g., databases with dates of coverage, contact with study authors to identify additional studies) in the search and date last searched.	2-3
Search	8	Present full electronic search strategy for at least one database, including any limits used, such that it could be repeated.	2
Study selection	9	State the process for selecting studies (i.e., screening, eligibility, included in systematic review, and, if applicable, included in the meta-analysis).	4-5
Data collection process	10	Describe method of data extraction from reports (e.g., piloted forms, independently, in duplicate) and any processes for obtaining and confirming data from investigators.	2
Data items	11	List and define all variables for which data were sought (e.g., PICOS, funding sources) and any assumptions and simplifications made.	3
Risk of bias in individual studies	12	Describe methods used for assessing risk of bias of individual studies (including specification of whether this was done at the study or outcome level), and how this information is to be used in any data synthesis.	2-4
Summary measures	13	State the principal summary measures (e.g., risk ratio, difference in means).	3
Synthesis of results	14	Describe the methods of handling data and combining results of studies, if done, including measures of consistency (e.g., I^2) for each meta-analysis.	3-4

PRISMA 2009 Checklist

Section/topic	#	Checklist item	Reported on page #
Risk of bias across studies	15	Specify any assessment of risk of bias that may affect the cumulative evidence (e.g., publication bias, selective reporting within studies).	3
Additional analyses	16	Describe methods of additional analyses (e.g., sensitivity or subgroup analyses, meta-regression), if done, indicating which were pre-specified.	4
**RESULTS**			
Study selection	17	Give numbers of studies screened, assessed for eligibility, and included in the review, with reasons for exclusions at each stage, ideally with a flow diagram.	4-5
Study characteristics	18	For each study, present characteristics for which data were extracted (e.g., study size, PICOS, follow-up period) and provide the citations.	6-7
Risk of bias within studies	19	Present data on risk of bias of each study and, if available, any outcome level assessment (see item 12).	4-17
Results of individual studies	20	For all outcomes considered (benefits or harms), present, for each study: (a) simple summary data for each intervention group (b) effect estimates and confidence intervals, ideally with a forest plot.	4-17
Synthesis of results	21	Present results of each meta-analysis done, including confidence intervals and measures of consistency.	4-17
Risk of bias across studies	22	Present results of any assessment of risk of bias across studies (see Item 15).	4-17
Additional analysis	23	Give results of additional analyses, if done (e.g., sensitivity or subgroup analyses, meta-regression [see Item 16]).	4-17
**DISCUSSION**			
Summary of evidence	24	Summarize the main findings including the strength of evidence for each main outcome; consider their relevance to key groups (e.g., healthcare providers, users, and policy makers).	17-18
Limitations	25	Discuss limitations at study and outcome level (e.g., risk of bias), and at review-level (e.g., incomplete retrieval of identified research, reporting bias).	19
Conclusions	26	Provide a general interpretation of the results in the context of other evidence, and implications for future research.	19
**FUNDING**			
Funding	27	Describe sources of funding for the systematic review and other support (e.g., supply of data); role of funders for the systematic review.	20

From: Moher D, Liberati A, Tetzlaff J, Altman DG, The PRISMA Group (2009). Preferred Reporting Items for Systematic Reviews and Meta-Analyses: The PRISMA Statement. PLoS Med 6(7): e1000097. doi:10.1371/journal.pmed1000097

For more information, visit: **www.prisma-statement.org**.

Supplementary Figure S1. Funnel plot (A) and sensitivity analysis (B) of studies that evaluated the effects of a very short abstinence period on seminal fluid volume in normozoospermic men

A

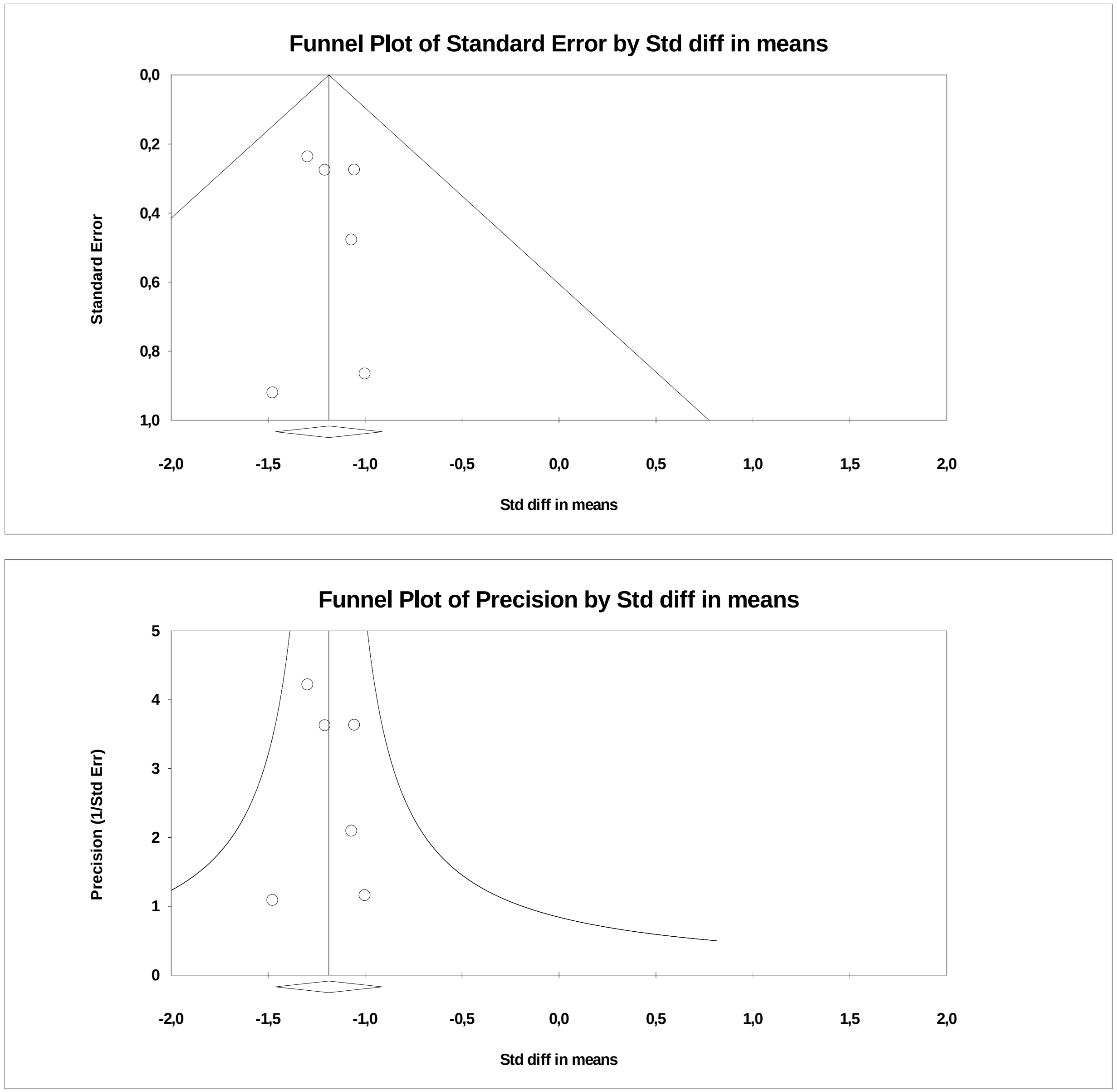

B

Sensitivity analysis

Study name	Statistics with study removed						
	Point	Standard error	Variance	Lower limit	Upper limit	Z-Value	p-Value
Alipour et al., 2017	-1,129	0,174	0,030	-1,469	-0,789	-6,502	0,000
Alipour et al., 2021	-1,180	0,163	0,026	-1,499	-0,862	-7,259	0,000
Hussein et al., 2008	-1,198	0,147	0,021	-1,485	-0,911	-8,175	0,000
Manna et al., 2020	-1,233	0,163	0,026	-1,552	-0,914	-7,580	0,000
Torres et al., 2016	-1,192	0,142	0,020	-1,470	-0,914	-8,395	0,000
Torres et al., 2016a	-1,180	0,142	0,020	-1,458	-0,902	-8,325	0,000
	-1,187	0,140	0,020	-1,461	-0,912	-8,471	0,000

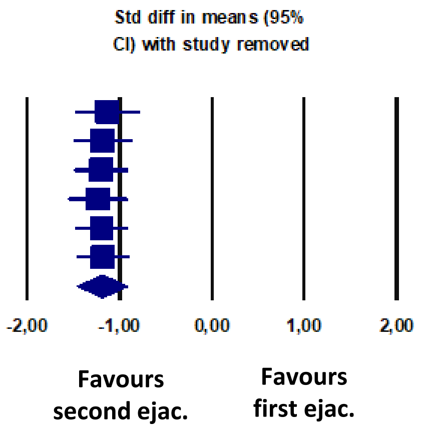

Supplementary Figure S2. Funnel plot (A) and sensitivity analysis (B) of studies that evaluated the effects of a very short abstinence period on seminal fluid volume in Oligo- and/or Astheno- and/or Terato- zoospermic men

A

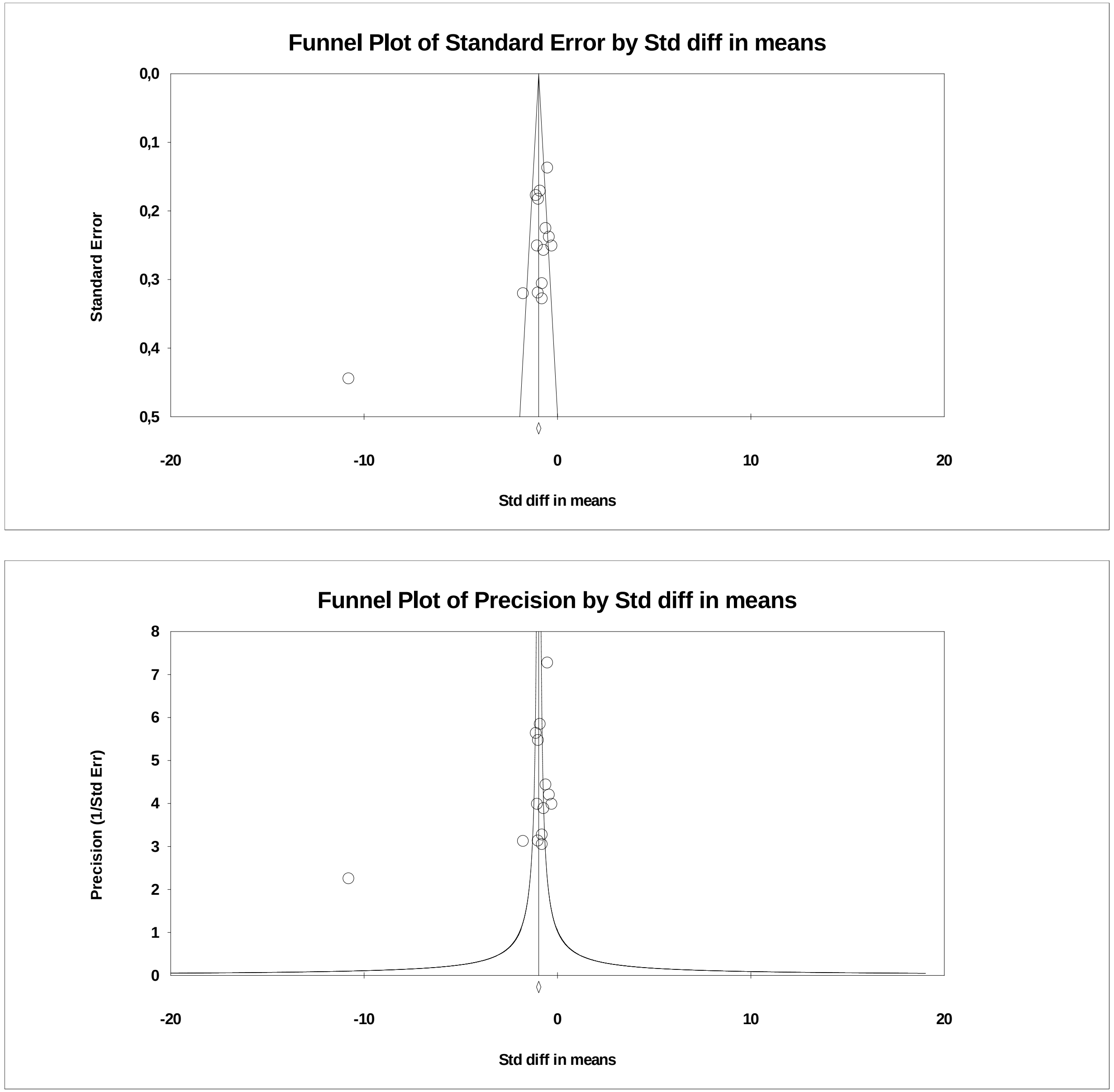

B

Sensitivity analysis

Study name	Point	Standard error	Variance	Lower limit	Upper limit	Z-Value	p-Value
	Statistics with study removed						
Bahadur et al., 2016	-0,955	0,064	0,004	-1,080	-0,830	-14,960	0,000
Barash et al., 1995	-1,009	0,062	0,004	-1,131	-0,887	-16,250	0,000
Bar-Hava et al., 2000	-1,081	0,067	0,004	-1,212	-0,950	-16,191	0,000
Ciotti et al., 2021	-0,983	0,064	0,004	-1,109	-0,857	-15,322	0,000
Hussein et al., 2008	-0,978	0,061	0,004	-1,098	-0,858	-16,001	0,000
Kulkarni et al., 2022	-0,970	0,064	0,004	-1,095	-0,845	-15,249	0,000
Manna et al., 2020	-0,967	0,062	0,004	-1,089	-0,846	-15,635	0,000
Ortiz et al., 2016	-0,987	0,062	0,004	-1,108	-0,866	-15,974	0,000
Patel et al., 2022	-1,000	0,062	0,004	-1,123	-0,878	-16,049	0,000
Ragheb et al., 2018	-0,789	0,061	0,004	-0,908	-0,670	-13,017	0,000
Scarselli et al., 2019	-0,971	0,061	0,004	-1,091	-0,851	-15,868	0,000
Sigiyan et al., 2008	-1,013	0,062	0,004	-1,134	-0,892	-16,375	0,000
Tur-Kaspa et al., 1994	-0,943	0,061	0,004	-1,063	-0,823	-15,414	0,000
Tur-Kaspa et al., 1994	-0,979	0,061	0,004	-1,099	-0,859	-15,978	0,000
	-0,972	0,060	0,004	-1,089	-0,854	-16,171	0,000

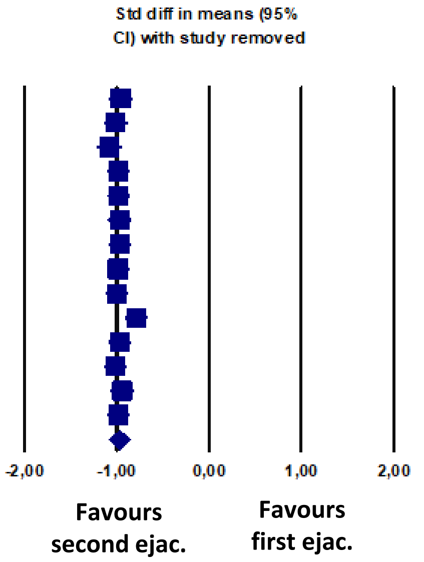

Supplementary Figure S3. Funnel plot (A) and sensitivity analysis (B) of studies that evaluated the effects of a very short abstinence period on sperm concentration in normozoospermic men

A

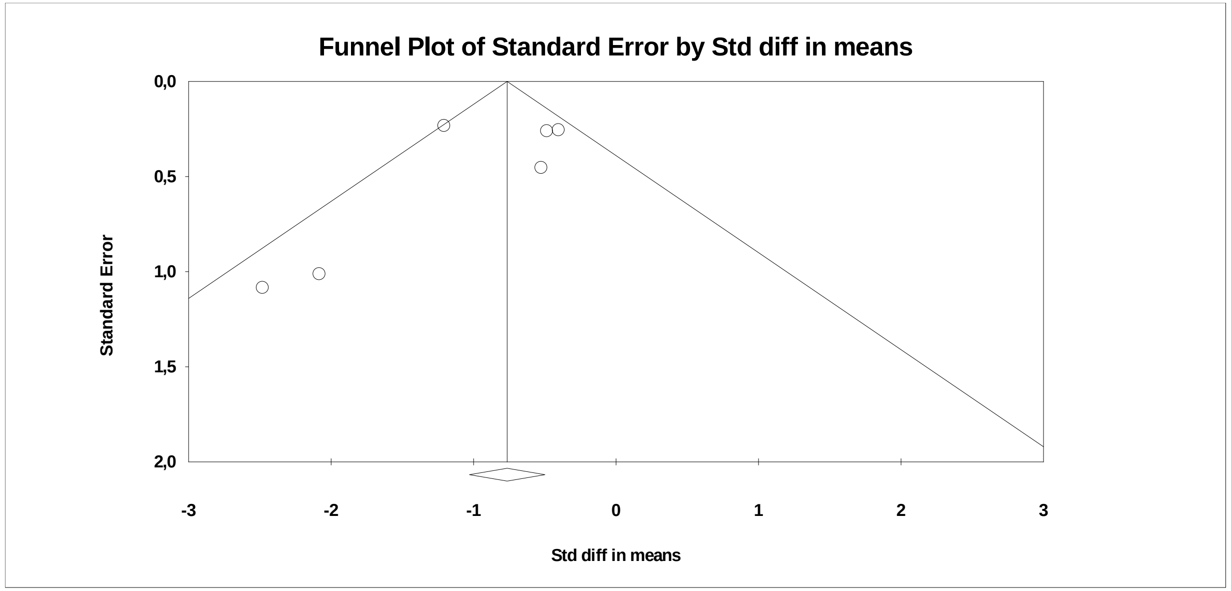

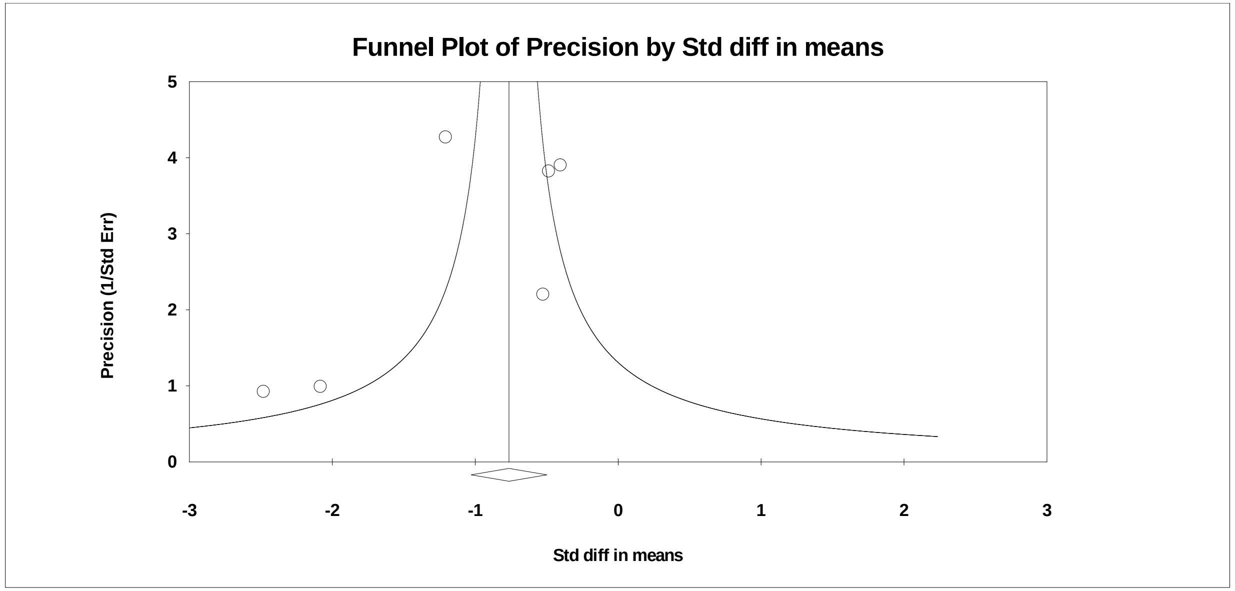

B

Sensitivity analysis

Study name	Statistics with study removed						
	Point	Standard error	Variance	Lower limit	Upper limit	Z-Value	p-Value
Alipour et al., 2017	-0,544	0,166	0,027	-0,869	-0,219	-3,282	0,001
Alipour et al., 2021	-0,904	0,159	0,025	-1,216	-0,592	-5,675	0,000
Hussein et al., 2008	-0,787	0,142	0,020	-1,065	-0,510	-5,556	0,000
Manna et al., 2020	-0,866	0,158	0,025	-1,176	-0,557	-5,482	0,000
Torre et al., 2016	-0,740	0,137	0,019	-1,008	-0,473	-5,422	0,000
Torre et al., 2016	-0,737	0,136	0,019	-1,004	-0,470	-5,405	0,000
	-0,764	0,135	0,018	-1,029	-0,499	-5,647	0,000

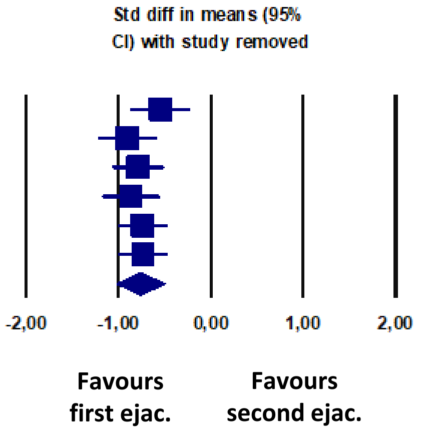

Supplementary Figure S4. Funnel plot (A) and sensitivity analysis (B) of studies that evaluated the effects of a very short abstinence period on sperm concentration in Oligo- and/or Astheno- and/or Terato- zoospermic men

A

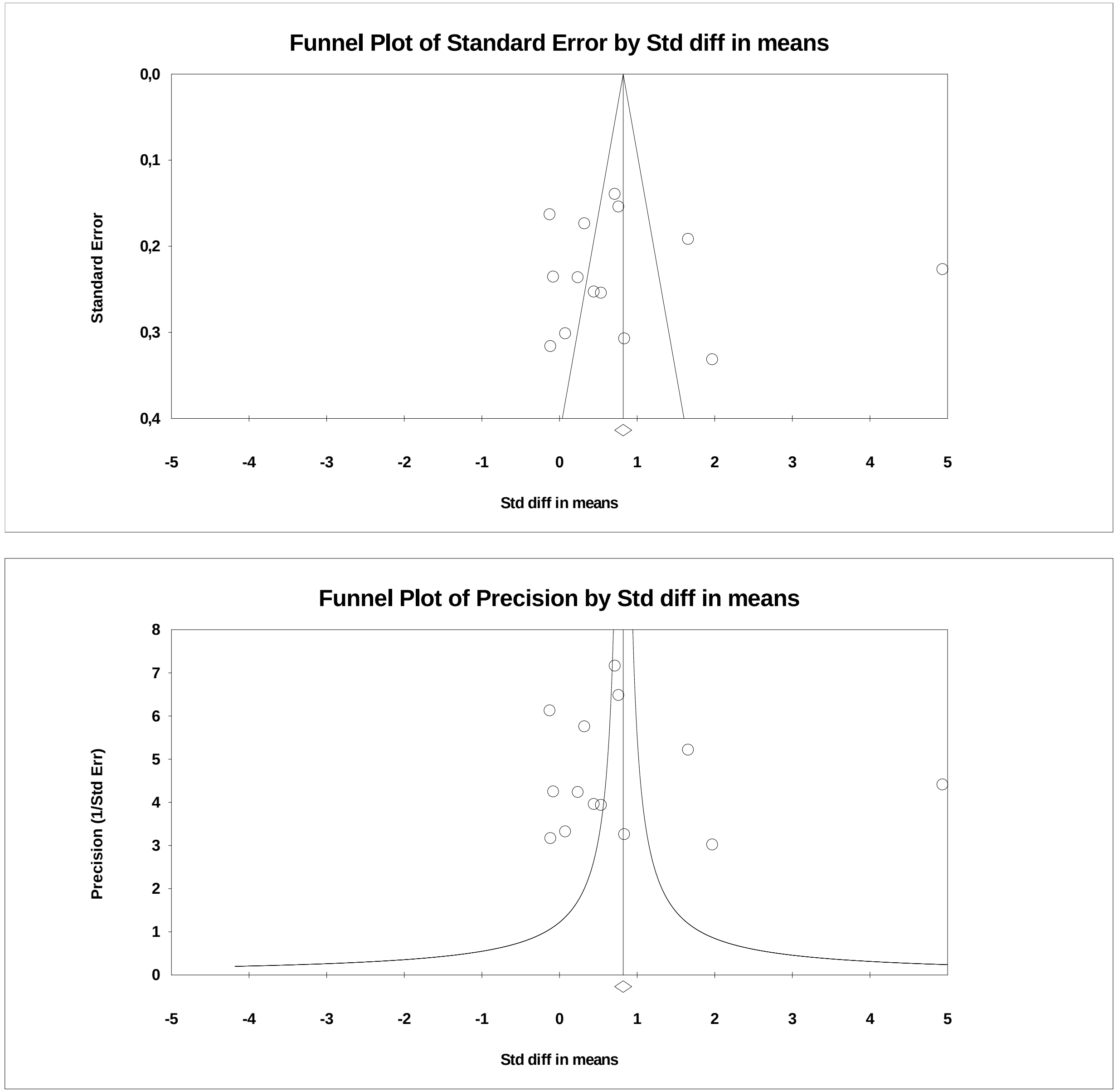

B

Sensitivity analysis

Study name	Statistics with study removed						
	Point	Standard error	Variance	Lower limit	Upper limit	Z-Value	p-Value
Bahadur et al., 2016	0,742	0,059	0,003	0,627	0,857	12,645	0,000
Barash et al., 1995	0,855	0,058	0,003	0,742	0,968	14,804	0,000
Barbagallo et al., 2021	0,829	0,060	0,004	0,711	0,947	13,762	0,000
Bar-Hava et al., 2000	0,840	0,061	0,004	0,720	0,960	13,715	0,000
Ciotti et al., 2021	0,946	0,060	0,004	0,829	1,063	15,841	0,000
Hussein et al., 2008	0,851	0,057	0,003	0,739	0,962	14,922	0,000
Kulkarmi et al., 2022	0,878	0,059	0,004	0,762	0,994	14,811	0,000
Manna et al., 2020	0,874	0,058	0,003	0,761	0,987	15,134	0,000
Ortiz et al., 2016	0,835	0,058	0,003	0,722	0,947	14,512	0,000
Ragheb et al., 2018	0,553	0,058	0,003	0,439	0,666	9,547	0,000
Scarselli et al., 2019	0,847	0,057	0,003	0,735	0,959	14,833	0,000
Sugiyam et al., 2008	0,840	0,058	0,003	0,727	0,952	14,594	0,000
Tur-Kaspa et al., 1994	0,786	0,057	0,003	0,675	0,898	13,816	0,000
Tur-Kaspa et al., 1994	0,820	0,057	0,003	0,708	0,932	14,366	0,000
	0,820	0,056	0,003	0,710	0,930	14,622	0,000

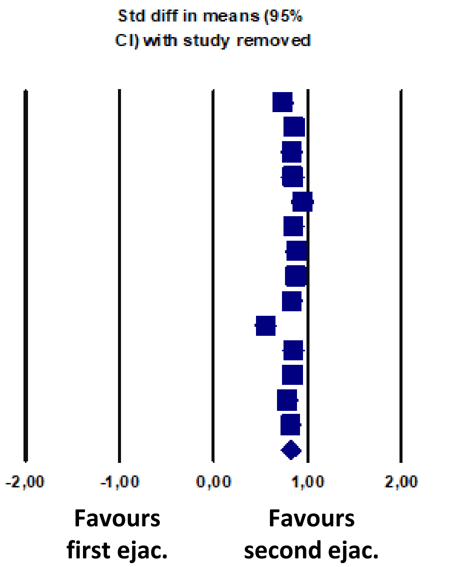

Supplementary Figure S5. Funnel plot (A) and sensitivity analysis (B) of studies that evaluated the effects of a very short abstinence period on sperm total motility in normozoospermic men

A

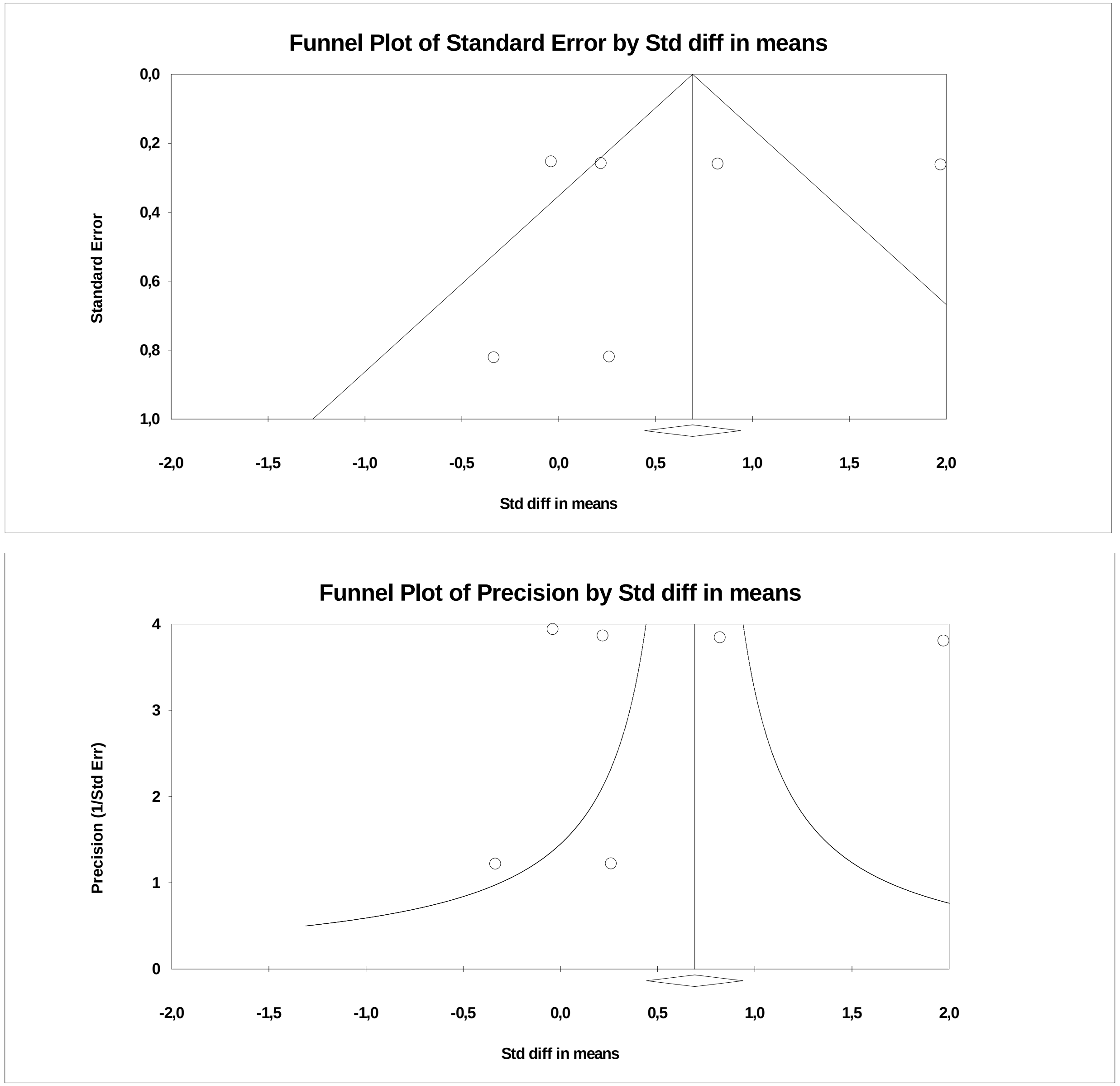

B

Sensitivity analysis

Study name	Statistics with study removed						
	Point	Standard error	Variance	Lower limit	Upper limit	Z-Value	p-Value
Alipour et al., 2017	0,306	0,144	0,021	0,023	0,589	2,123	0,034
Alipour et al., 2021	0,931	0,146	0,021	0,645	1,217	6,389	0,000
Manna et al., 2020	0,839	0,145	0,021	0,555	1,123	5,791	0,000
Sugiyam et al., 2008	0,651	0,145	0,021	0,367	0,934	4,500	0,000
Torres et al., 2016	0,716	0,128	0,016	0,465	0,967	5,597	0,000
Torres et al., 2016	0,702	0,128	0,016	0,451	0,952	5,485	0,000
	0,691	0,126	0,016	0,443	0,939	5,468	0,000

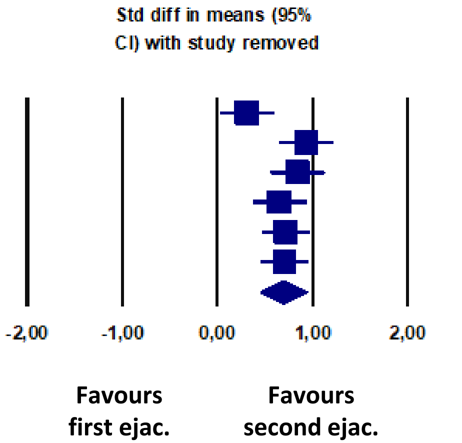

Supplementary Figure S6. Funnel plot (A) and sensitivity analysis (B) of studies that evaluated the effects of a very short abstinence period on sperm total motility in Oligo- and/or Astheno- and/or Terato- zoospermic men

A

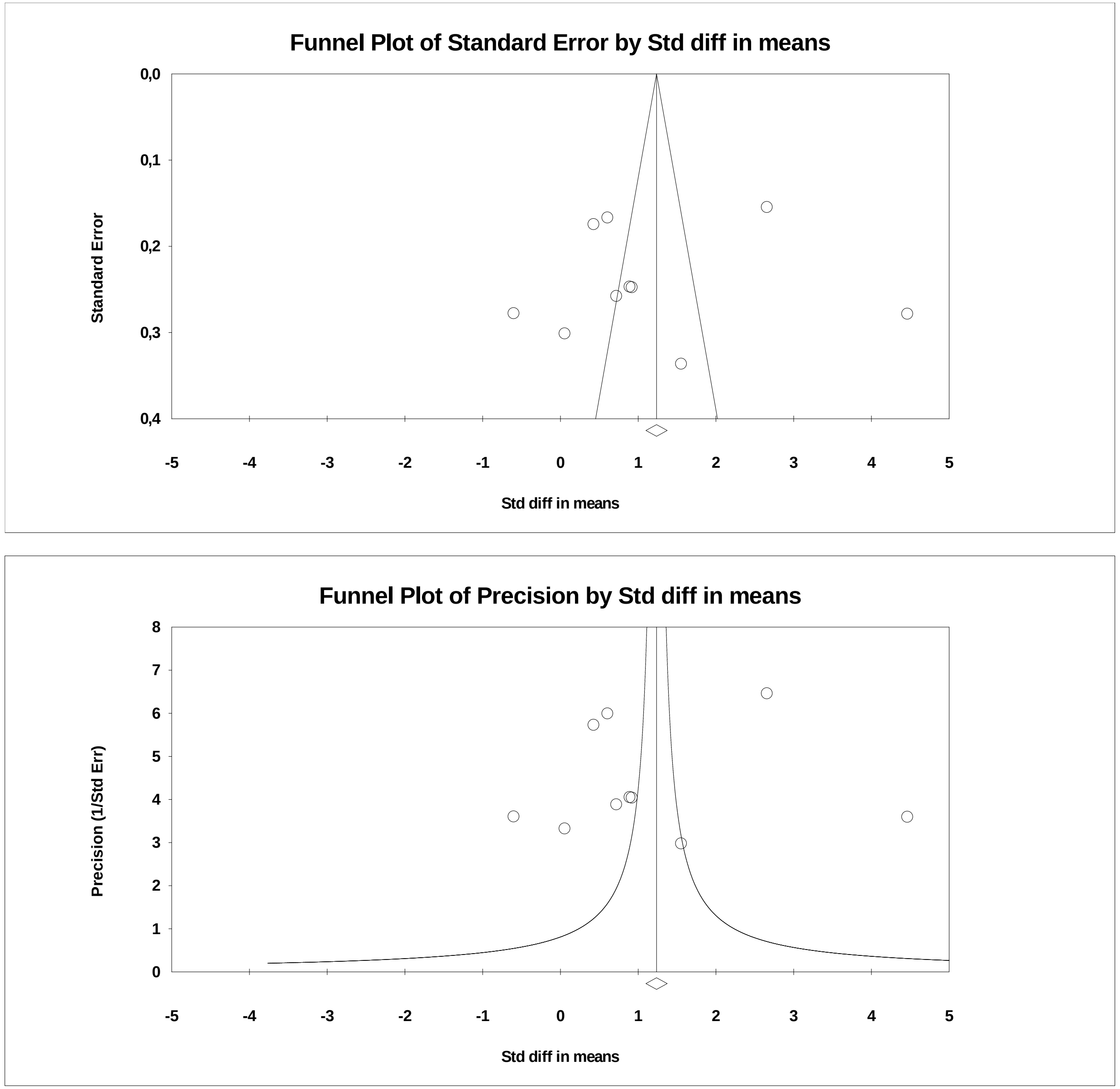

B

Sensitivity analysis

Study name	Statistics with study removed						
	Point	Standard error	Variance	Lower limit	Upper limit	Z-Value	p-Value
Barash et al., 1995	1,266	0,073	0,005	1,123	1,409	17,319	0,000
Barbagallo et al., 2021	1,018	0,072	0,005	0,876	1,160	14,055	0,000
Ciotti et al., 2021	1,370	0,077	0,006	1,219	1,521	17,739	0,000
Kulkaemi et al., 2022	1,391	0,077	0,006	1,241	1,541	18,172	0,000
Manna et al., 2000	1,264	0,073	0,005	1,120	1,407	17,288	0,000
Ortiz et al., 2015	1,277	0,073	0,005	1,134	1,420	17,533	0,000
Ragheb et al., 2018	0,870	0,079	0,006	0,716	1,024	11,064	0,000
Scarselli et al., 2019	1,304	0,072	0,005	1,162	1,445	18,085	0,000
Tur-Kaspa et al., 1994	1,361	0,072	0,005	1,219	1,503	18,782	0,000
Tur-Kaspa et al., 1994	1,222	0,072	0,005	1,081	1,362	17,045	0,000
	1,236	0,070	0,005	1,099	1,374	17,636	0,000

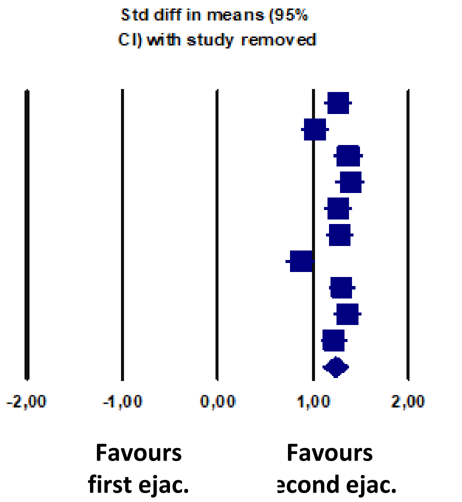

Supplementary Figure S7. Funnel plot (A) and sensitivity analysis (B) of studies that evaluated the effects of a very short abstinence period on sperm progressive motility in normozoospermic men

A

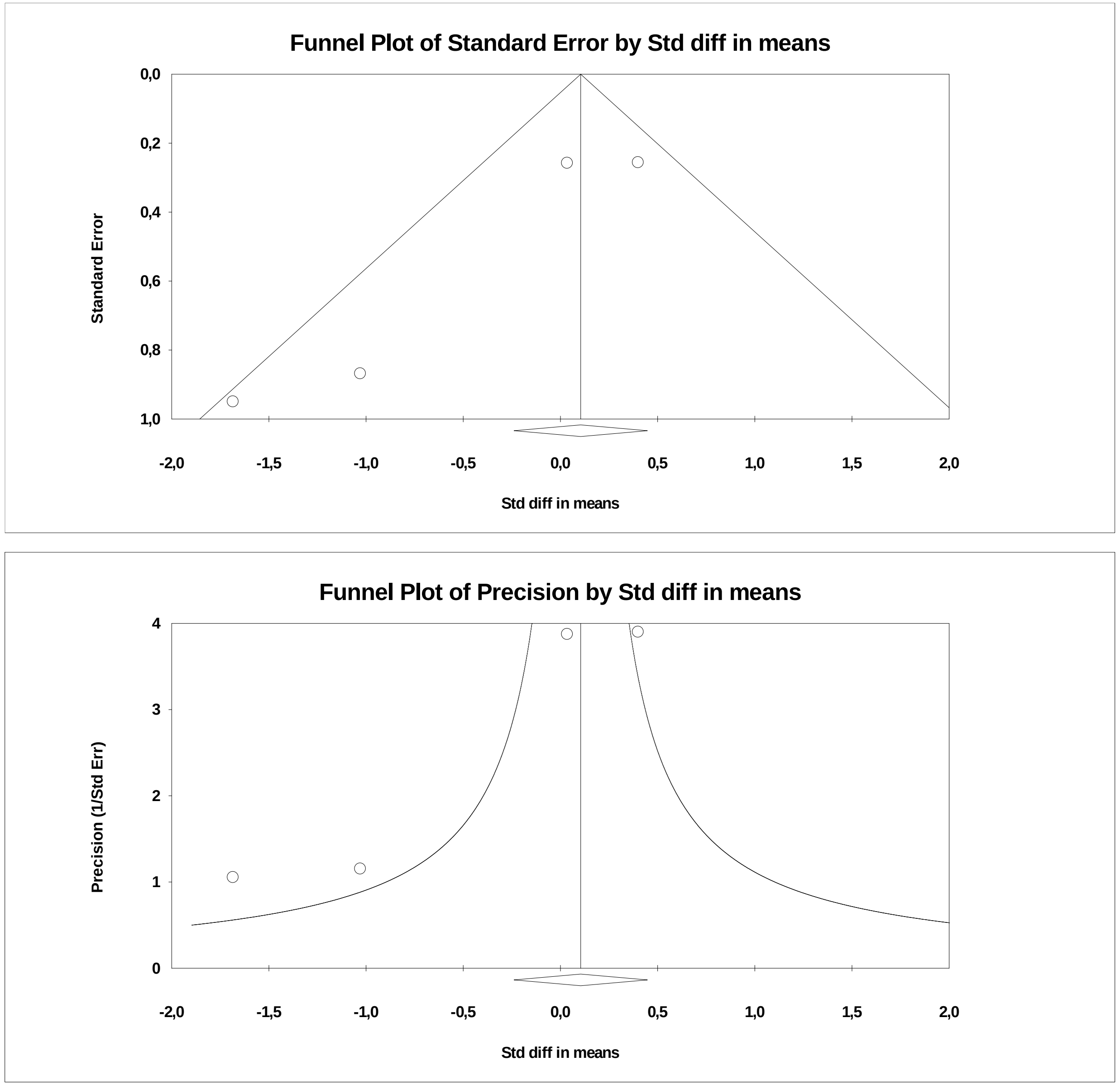

B

Sensitivity analysis

Study name	Statistics with study removed						
	Point	Standard error	Variance	Lower limit	Upper limit	Z-Value	p-Value
Alipour et al., 2021	-0,154	0,240	0,057	-0,624	0,315	-0,644	0,520
Manna et al., 2020	0,162	0,238	0,057	-0,305	0,629	0,681	0,496
Torres et al., 2016	0,167	0,178	0,032	-0,182	0,516	0,938	0,348
Torres et al., 2016	0,152	0,179	0,032	-0,198	0,503	0,851	0,394
	0,104	0,175	0,031	-0,239	0,447	0,595	0,552

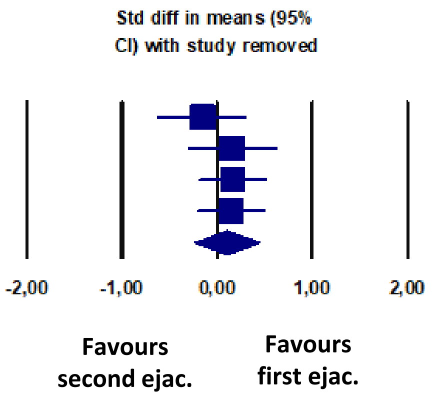

Supplementary Figure S8. Funnel plot (A) and sensitivity analysis (B) of studies that evaluated the effects of a very short abstinence period on sperm progressive motility in Oligo- and/or Astheno- and/or Terato- zoospermic men

A

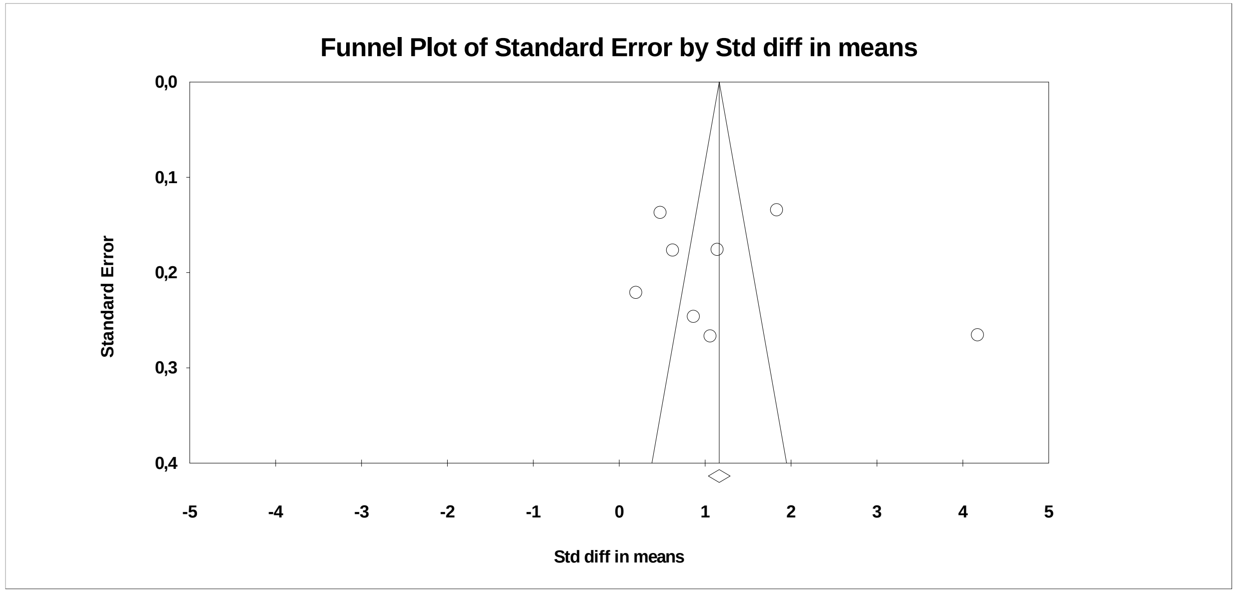

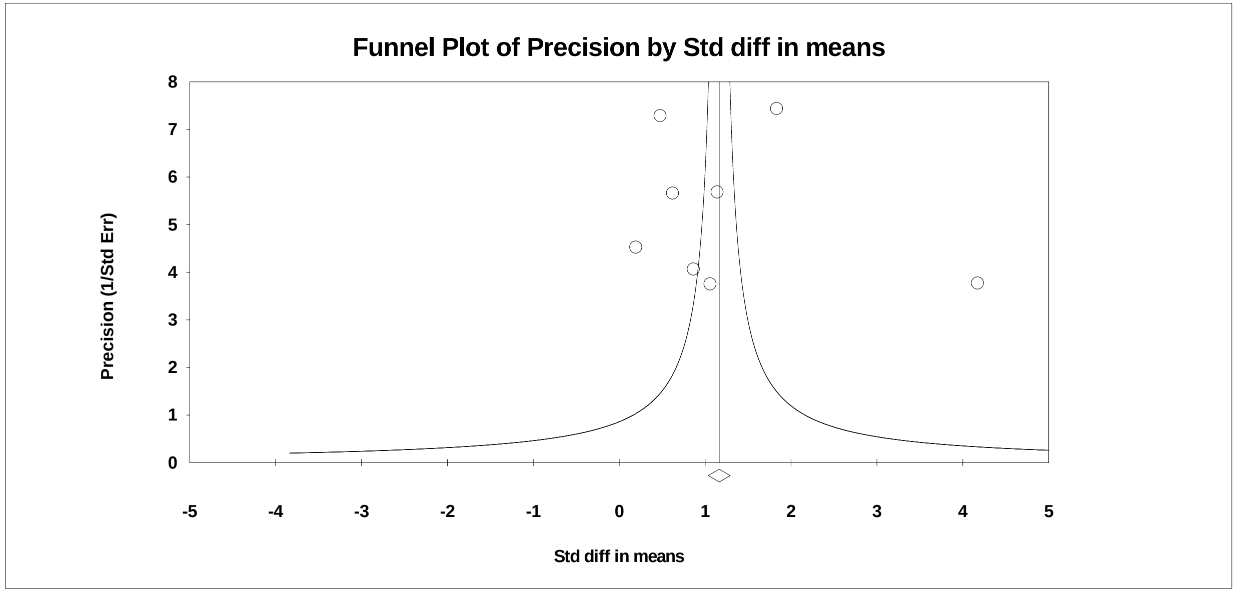

B

Sensitivity analysis

Study name	Point	Standard error	Variance	Lower limit	Upper limit	Z-Value	p-Value
	Statistics with study removed						
Barbagallo et al., 2021	0,973	0,067	0,004	0,842	1,105	14,541	0,000
Bar-Hava et al., 2000	1,360	0,074	0,005	1,216	1,505	18,471	0,000
Ciotti et al., 2021	1,167	0,070	0,005	1,030	1,304	16,717	0,000
Kulkarmi et al., 2022	1,248	0,070	0,005	1,111	1,385	17,883	0,000
Manna et al., 2020	1,186	0,067	0,005	1,054	1,318	17,631	0,000
Ortiz et al., 2016	1,171	0,067	0,004	1,040	1,302	17,494	0,000
Patel et al., 2022	1,255	0,068	0,005	1,122	1,388	18,485	0,000
Ragheb et al., 2018	0,960	0,074	0,005	0,815	1,105	12,957	0,000
	1,164	0,065	0,004	1,037	1,292	17,937	0,000

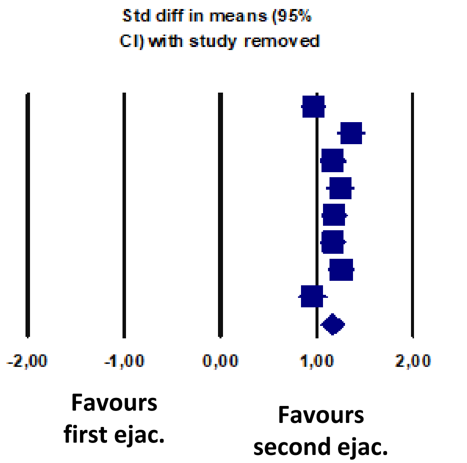

Supplementary Figure S9. Funnel plot (A) and sensitivity analysis (B) of studies that evaluated the effects of a very short abstinence period on sperm morphology in Oligo- and/or Astheno- and/or Terato- zoospermic men

A

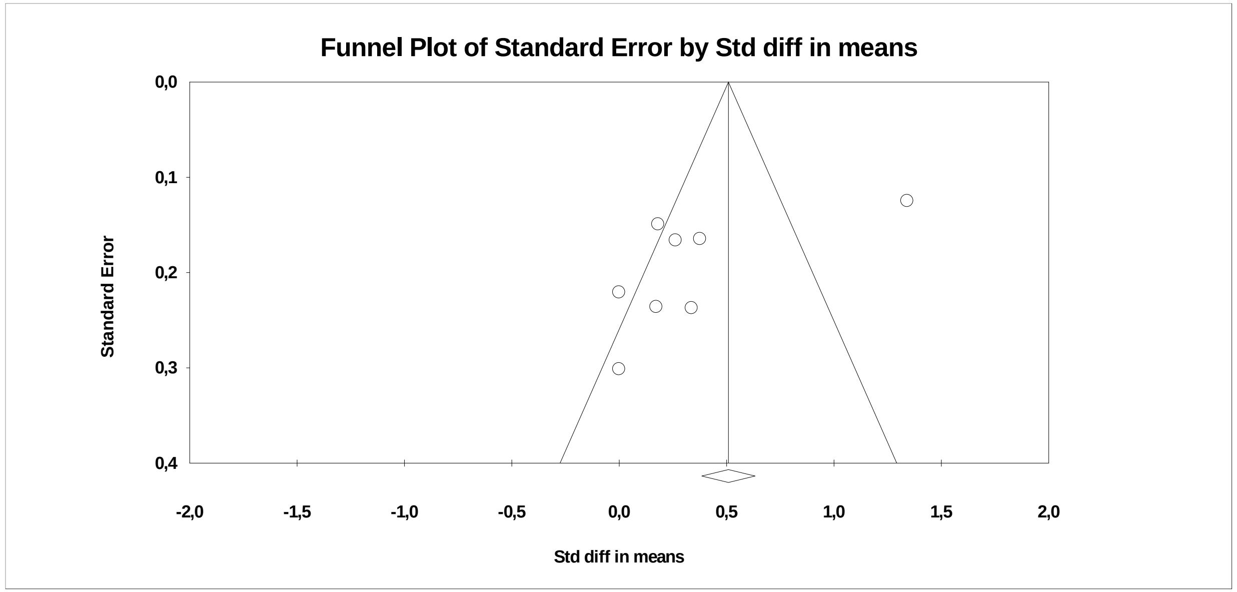

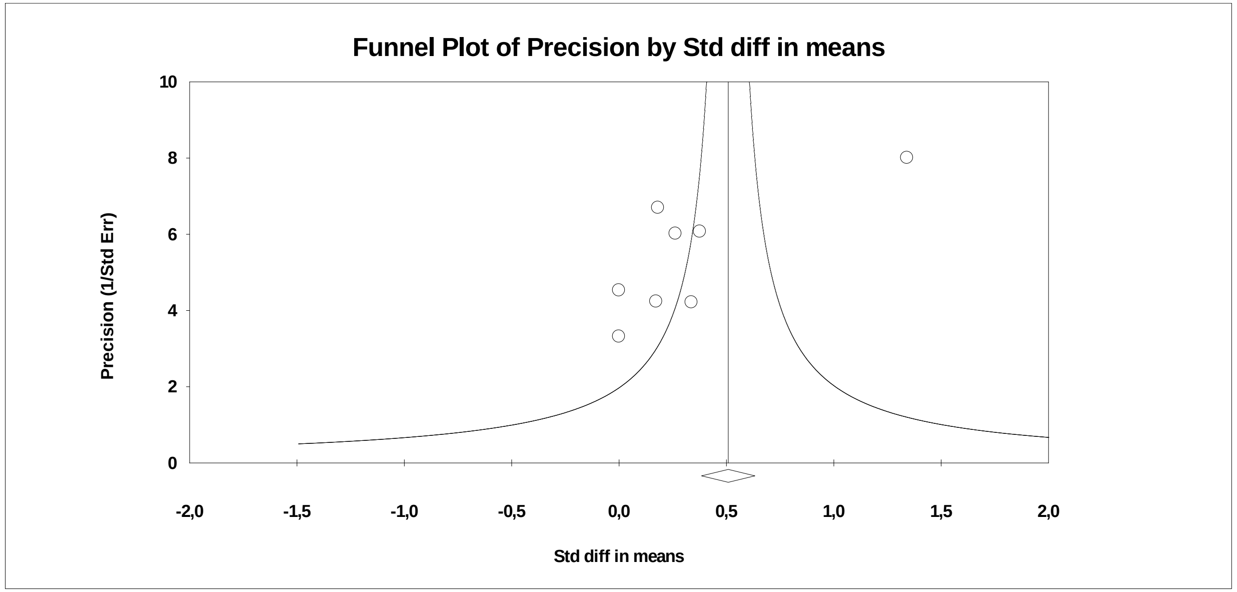

B

Sensitivity analysis

Study name	Statistics with study removed						
	Point	Standard error	Variance	Lower limit	Upper limit	Z-Value	p-Value
Bahadur et al., 2016	0,550	0,069	0,005	0,416	0,684	8,025	0,000
Barash et al., 1995	0,534	0,066	0,004	0,405	0,663	8,124	0,000
Barbagallo et al., 2021	0,580	0,070	0,005	0,443	0,717	8,289	0,000
Ciotti et al., 2021	0,531	0,069	0,005	0,397	0,666	7,739	0,000
Manna et al., 2020	0,521	0,066	0,004	0,393	0,650	7,931	0,000
Patel et al., 2022	0,554	0,066	0,004	0,424	0,684	8,375	0,000
Ragheb et al., 2018	0,220	0,074	0,005	0,076	0,364	2,989	0,003
Scarselli et al., 2019	0,532	0,065	0,004	0,405	0,659	8,206	0,000
	0,508	0,063	0,004	0,384	0,633	8,023	0,000

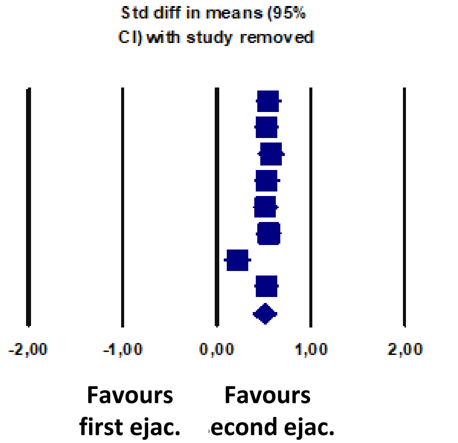

Supplementary Figure S10. Funnel plot (A) and sensitivity analysis (B) of studies that evaluated the effects of a very short abstinence period on sperm DNA fragmentation in normozoospermic men

A

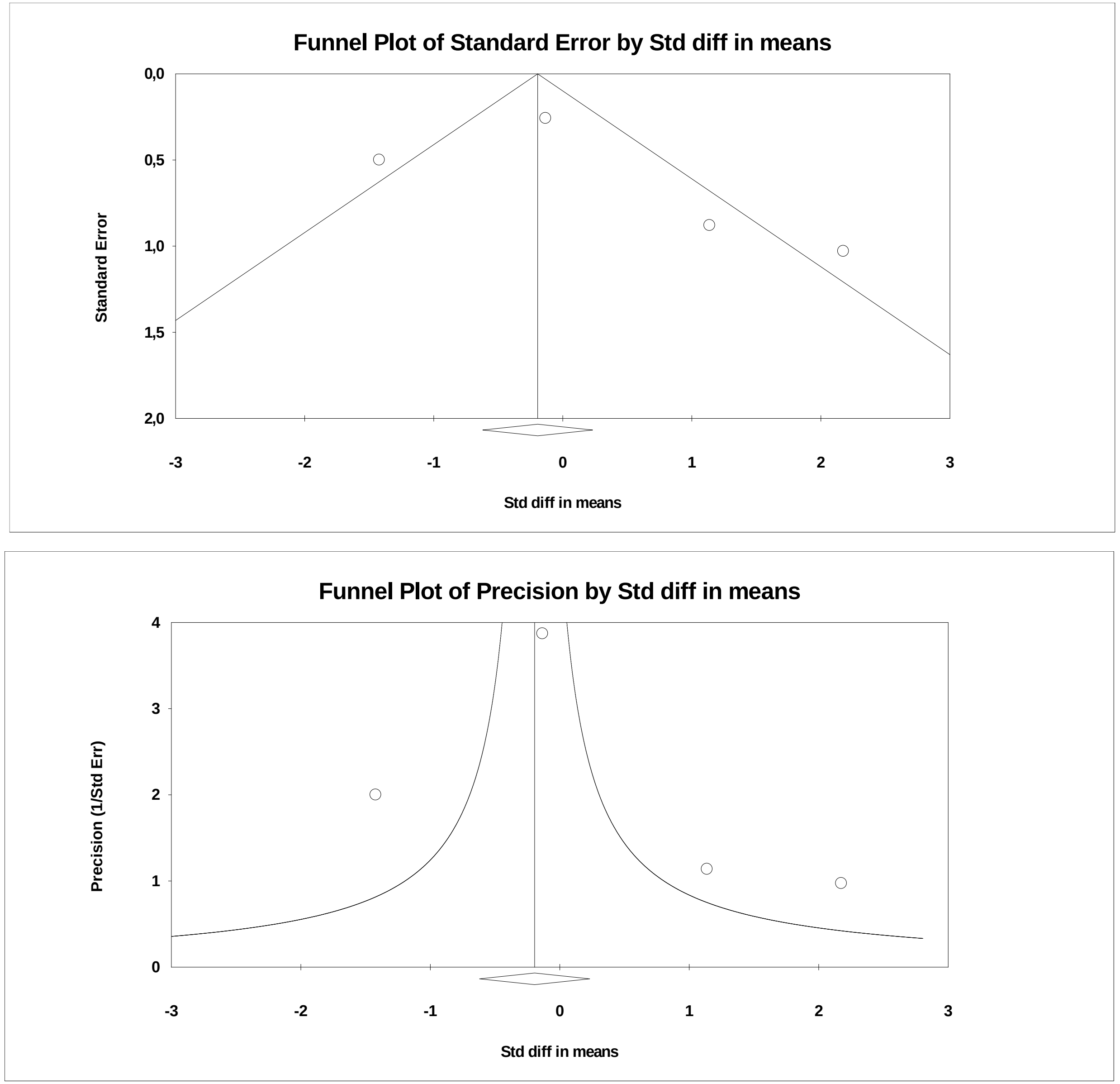

B

Sensitivity analysis

Study name	Statistics with study removed						
	Point	Standard error	Variance	Lower limit	Upper limit	Z-Value	p-Value
Hussein et al., 2008	0,090	0,241	0,058	-0,382	0,563	0,374	0,709
Manna et al., 2020	-0,345	0,401	0,161	-1,131	0,440	-0,862	0,389
Torres et al., 2016	-0,305	0,222	0,049	-0,740	0,131	-1,371	0,170
Torres et al., 2016	-0,281	0,224	0,050	-0,720	0,158	-1,253	0,210
	-0,194	0,217	0,047	-0,620	0,231	-0,895	0,371

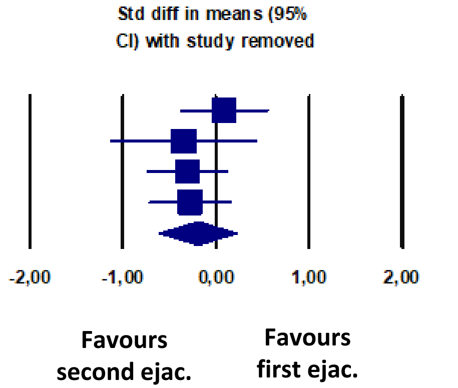

Supplementary Figure S11. Funnel plot (A) and sensitivity analysis (B) of studies that evaluated the effects of a very short abstinence period on sperm DNA fragmentation in Oligo- and/or Astheno- and/or Terato- zoospermic men

A

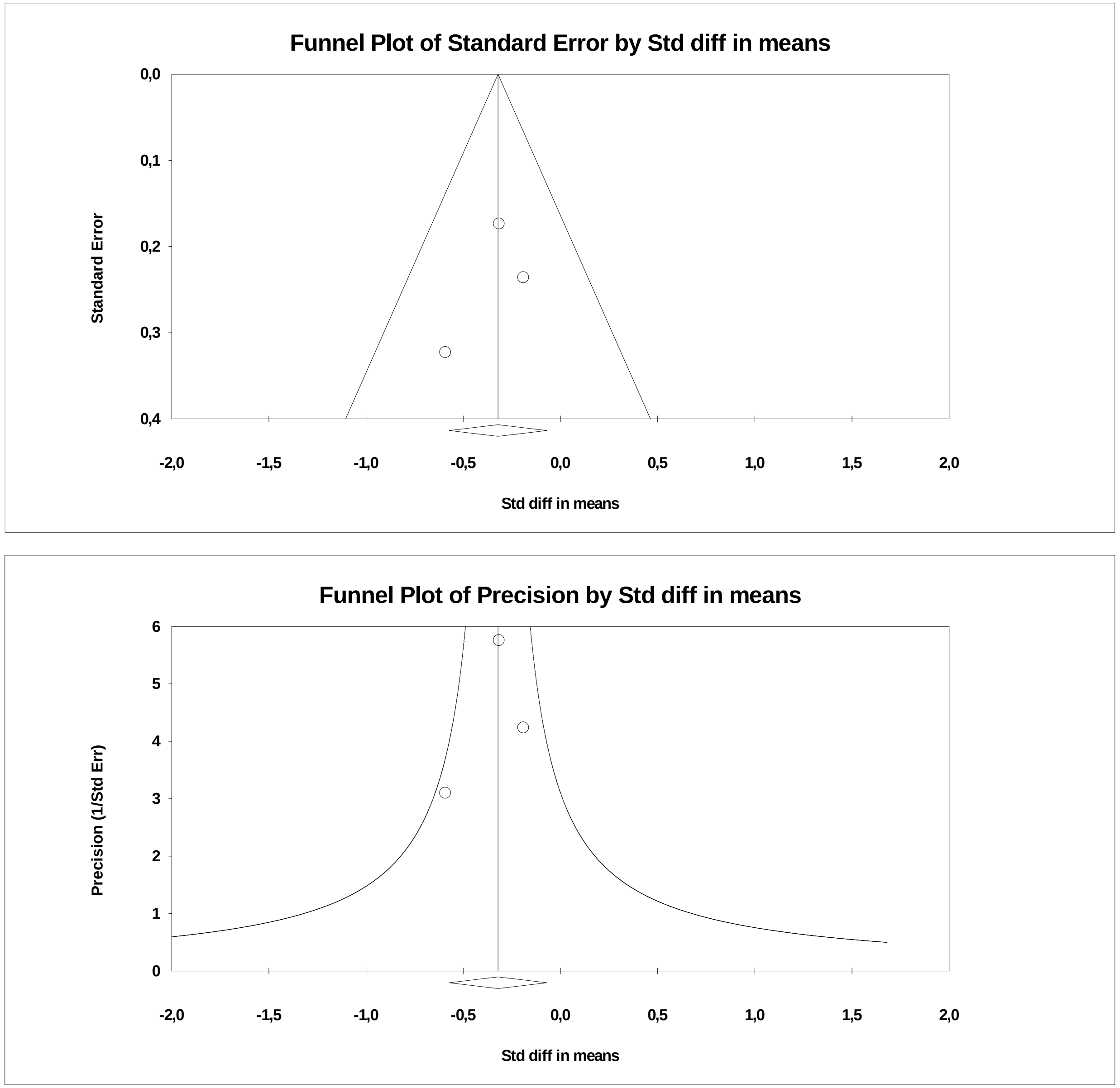

B

Sensitivity analysis

Study name	Statistics with study removed						
	Point	Standard error	Variance	Lower limit	Upper limit	Z-Value	p-Value
Hussein et al., 2008	-0,270	0,140	0,020	-0,545	0,004	-1,931	0,053
Kulkarmi et al., 2022	-0,329	0,191	0,036	-0,703	0,045	-1,725	0,084
Manna et al., 2020	-0,377	0,153	0,023	-0,677	-0,076	-2,460	0,014
	-0,321	0,128	0,017	-0,573	-0,069	-2,499	0,012

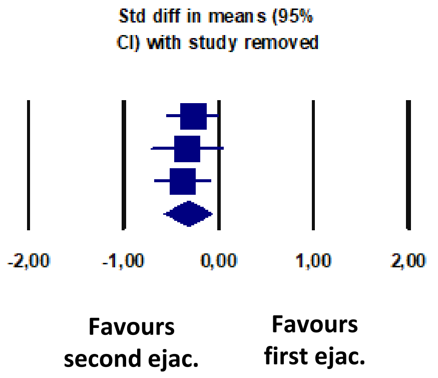

Supplement: Supplementary file 1 [file jcm-11-07303-s001.zip › jcm-2076644-supplementary.pdf]
